# A stochastic framework to model axon interactions within growing neuronal populations

**DOI:** 10.1371/journal.pcbi.1006627

**Published:** 2018-12-03

**Authors:** Agustina Razetti, Caroline Medioni, Grégoire Malandain, Florence Besse, Xavier Descombes

**Affiliations:** 1 Université Côte d’Azur, INRIA, CNRS, I3S, Nice, France; 2 Université Côte d’Azur, CNRS, Inserm, iBV, Nice, France; The University of Queensland, AUSTRALIA

## Abstract

The confined and crowded environment of developing brains imposes spatial constraints on neuronal cells that have evolved individual and collective strategies to optimize their growth. These include organizing neurons into populations extending their axons to common target territories. How individual axons interact with each other within such populations to optimize innervation is currently unclear and difficult to analyze experimentally *in vivo*. Here, we developed a stochastic model of 3D axon growth that takes into account spatial environmental constraints, physical interactions between neighboring axons, and branch formation. This general, predictive and robust model, when fed with parameters estimated on real neurons from the *Drosophila* brain, enabled the study of the mechanistic principles underlying the growth of axonal populations. First, it provided a novel explanation for the diversity of growth and branching patterns observed *in vivo* within populations of genetically identical neurons. Second, it uncovered that axon branching could be a strategy optimizing the overall growth of axons competing with others in contexts of high axonal density. The flexibility of this framework will make it possible to investigate the rules underlying axon growth and regeneration in the context of various neuronal populations.

## Author summary

Understanding how neuronal cells establish complex circuits with specific functions within a developing brain is a major current challenge. Over the last past years, enormous progress has been done to precisely resolve brain anatomy and to dissect the mechanisms controlling the establishment of precise neuronal networks. However, due to the extreme complexity of the brain, it is still experimentally difficult to investigate *in vivo* how neurons interact with each other and with their physical environments to innervate target territories during development. Here, we have developed a framework that integrates a dynamic 3D mathematical model of single axonal growth with parameters estimated from neurons grown *in vivo* and simulations of entire populations of growing axons. The emergent properties of our model enable the study of the mechanistic principles underlying the growth of axonal population in developing brains. Specifically, our results highlight the impact of mechanical interactions on both individual and collective axon growth, and uncover how branching regulate this process.

## Introduction

*In vivo*, neurons extend processes that must navigate through the extremely dense and complex environment of developing brains to find their targets and assemble into functional networks. This environment provides the diffusible extracellular cues that guide neuronal processes to their destination by orienting their growth and promoting their extension or retraction [1–6]. As revealed by recent work, axons are not only capable of sensing and responding to external chemical signals, but are also guided by local changes in the mechanical properties of their surrounding [7–9]. Beyond providing chemical or mechanical cues orienting axon navigation, the confined and crowded environment of the brain also imposes spatial constraints on the organization and growth of neuronal projections. Thus, strategies to optimize growth in the context of increasing numbers of neurons have been developed across evolution. These include organization of neurons into populations that coordinate to grow and innervate target territories [10, 11]. Although studies have revealed that inter-neuron coordination and interactions are particularly important in the context of a developing population [10, 12–15], axon growth has so far been mainly studied *in vitro* on isolated neurons, or *in vivo* on whole populations of neurons. However, to fully understand population growth, one needs to understand the behavior of single constituent neurons, and how they interact and influence themselves to produce global growth. This is experimentally not trivial, as tools to reproducibly visualize and manipulate axon-axon interactions *in vivo* in populations of growing axons are either lacking or heavy to implement.

To overcome these difficulties, we have developed a 3D dynamic mathematical framework that generates simulations yielding realistic single cell morphologies and accurately reproduces the process of axon growth in a population context. Although models taking into account different aspects of axon competition have been described [16–21], 3D models considering spatial constraints and mechanical neuron-neuron interactions are so far rare [22–25]. Torben-Nielsen and De Schutter, for example, elaborated a framework for context-aware neuron development where growth rules are mainly phenomenological [23]. Zubler et al. proposed a model of neuron growth based on physical forces between objects and diffusion of substances through the extracellular domain [24]. Vanherpe et al. proposed a framework for the development of non-intersecting tubular-like structures in confined spaces, which highlighted the dependence of axon elongation and final morphology on spatial boundaries and axonal density [25]. Together, these models stressed the importance of considering space-embedded processes and interactions with the cellular environment when studying neuronal morphologies. However, they have not, or could not, use parameters estimated from real data, and did not showcase explicative or predictive aspects of their models.

In this work, we developed a flexible framework that can integrate data from biological samples with mathematical modeling to uncover the principles underlying axon growth in a population context. First, we proposed a 3D stochastic model for the growth of individual axons which relies on parameters that can be estimated from data. This model can be implemented with branch formation, and applied to the simulation of individual axons. Second, we simulated the growth of populations of axons, letting them grow simultaneously, in a spatially constrained environment where they compete for space and change their path when encountering obstacles.

To test our model on biological data, we took advantage of a database of confocal images representing single mature axons grown in the context of *Drosophila* brains. Strikingly, our framework generated a range of growth and arborization patterns similar to that observed in *Drosophila* brains, when implemented with parameters estimated from *in* *vivo* data, providing a mechanistic explanation for the origin of observed morphological diversity. Furthermore, modulating the capacity of axons to form branches influenced axon growth. At the population level, branching axons grew more efficiently than non-branching ones. At the single-cell level, non-branching axons were out-competed by branching ones, generating defective growth profiles similar to those observed *in* *vivo* upon inactivation of *imp*, a gene known for its role in axon growth and branching. While the importance of branching has so far been mostly considered in the context of establishing connections with partners [26, 27], our results thus suggest that branching may also be a strategy adopted during development to overcome spatial competition and optimize growth in contexts of high axonal density. Together, the simple, explicative and predictive framework we have developed enables mechanistic studies of the principles governing the collective growth of axons in realistic spatially constrained environments.

## Results

Here, we propose a simulation framework for the collective growth of axonal extensions. Individual 3D axonal morphologies are generated by a mathematical model for single axonal path generation, and an algorithmic implementation of branch occurrence. Time is also implemented algorithmically, and interactions between individuals are considered *via* volume exclusion. Parameters can be estimated from real data, which allows the model to be not only generative, but also explicative of key aspects of the growth process, and predictive.

### Modeling individual axonal trees

#### Axonal paths

**Fig 1:**
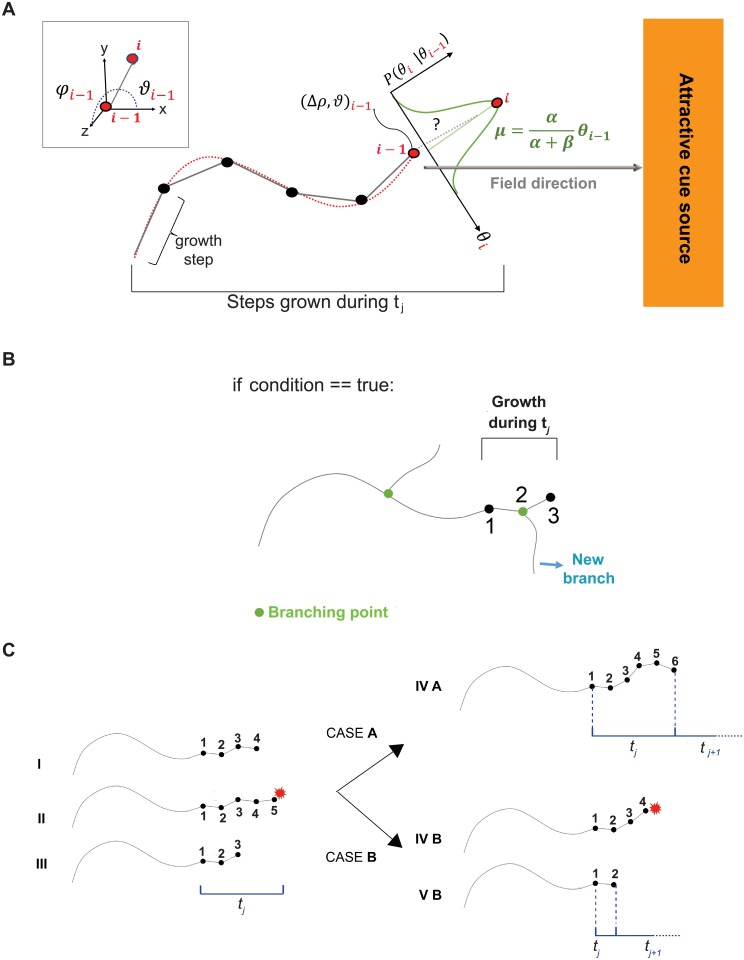
3D mathematical model of individual axon growth. (A) The axon elongates step by step (grey segments), each step being delimited by the current point in space *i* and the previous one, *i* − 1. Each *i*^*th*^ new step is described by its spherical coordinates (Δ*ρ*, *ϑ*, *φ*) (represented in 2D for simplicity; see upper left corner for a 3D view), and placed after the previous position *i* − 1. The exact position of the *i*^*th*^ point is defined (in 2D) by $\theta_{xy}^{i}=f\left(\phi_{x,y}^{i}\right)$, which is drawn from a conditional Normal probability distribution (in green). The most probable value (mean of the distribution (*μ*)) considers the directions of the last step *i* − 1 and of the attractive field, each contribution weighted by *α* and *β* respectively. For each time unit *t*_*j*_, a maximum number of steps (*n*_*max*_) is allowed. (B) Algorithmic implementation of branch generation. During *t*_*j*_, a certain number of growth steps are performed (3 in this schematic representation). At the end of *t*_*j*_, a certain “condition” is evaluated and, if true, a branch point is placed at the axon tip, or at any other step performed during *t*_*j*_ (step 2). (C) Step by step axon growth scheme during *t*_*j*_ considering physical interactions. The axon elongates of *n* steps (I), until it encounters a mechanical constraint (II) and retracts (III). During the same time interval *t*_*j*_, it will re-try to reach *n*_*max*_ steps (6 in this example). If no other mechanical constraint is encountered (CASE A), the axon will advance to reach *n*_*max*_ steps. If, on the contrary, another mechanical constraint is encountered (CASE B), the axon will retract again and stop its growth until the next time point (*t*_*j*+1_). In this case, the total growth during *t*_*j*_ is then smaller than *n*_*max*_ steps.

Axonal paths are modeled in 3D as the successive addition of discrete segments of fixed length, where the orientation of each new step follows a Markov chain that depends on the orientation of the previous step (Markov property), and the directionality of the attractive field (Fig 1A). This can also be expressed as a persistent (Markov property) biased (external field) random walk, as described in [28]. Our axonal path model (Eq 1) depends mainly on the following parameters: *α*, representing axon rigidity (axon capacity to bend, referred to as the persistence parameter) and *β*, representing the attraction to the external field (*ψ*) or bias. Other variables are also considered such as the step size Δ*ρ* and the dynamic parameter *n*_*max*_, which sets the maximum number of steps an axon grows during a time unit *t*_*j*_ and thus the maximum growth speed (*v*_*max*_) (Table 1). The external attractive field *ψ* should be proposed depending on the specific axon type to model. It may be designed as a simple uniform field parallel to the *x* axis (as in Fig 1 and S1 Fig), or acquire more complex morphologies, varying in function of space or time. Δ*ρ* defines the spatial resolution of the model, and can be fix or dynamic. In this work, Δ*ρ* was considered fix and chosen according to data sampling.

**Table 1:** Summary of model parameters and variables.

Symbol	Significance
**Morphological Parameters**	
*α*	Axon rigidity
*β*	Axon external attraction
Δ*ρ*	Step size
*ψ*	External attractive field
*P*_*b*_	Branch point generation probability in *t*_*j*_
*ω*	Branch initial angle distribution
λ_*b*_	Poisson parameter for branch distance
*b*_*l*_	Maximum branch order
*d*	Axon diameter
*Num*_*ax*_	Number of axons
**Dynamic Parameters**	
*n*_*max*_	Maximum number of steps grown in *t*_*j*_
*n*_*r*_	Retraction rate
counter_max_	Maximum value of trials upon interaction
**Spatial Parameters**	
*X*_*max*_	Maximum traveled distance
*ξ*	Growth cavity
**Variables**	
*t*_*j*_	Time point
*ϕ*_*i*_	Step angle
*θ*_*i*_	Model angle variable
counter	Number of trials upon interaction per growing tip

We use spherical coordinates (Δ*ρ*, *ϑ*, *φ*), where *ϑ* represents the angle in the xy plane and *φ* the elevation along the z axis. Each new vector step is placed after the previous one. Because the step size Δ*ρ* is constant, the model is reduced to two variables (*ϑ*, *φ*). Moreover, we consider that both angles are independent, reducing the full model to two sub-models with only one variable each: *ϑ*/*φ*.

The probability distribution of the *i*^*th*^ axonal tip Cartesian position (*x*, *y*, *z*)_*i*_ knowing the previous one (*x*, *y*, *z*)_*i*−1_, is thus directly defined by the probability distribution of (*ϑ*_*i*_, *φ*_*i*_) conditioned by (*ϑ*_*i*−1_, *φ*_*i*−1_), and defined by

$$\begin{array}{c} P\left(\theta_{i}|\theta_{i-1}\right)\propto exp-[\alpha {\left(\theta_{i}-\theta_{i-1}\right)}^{2}+\beta {\left(\theta_{i}\right)}^{2}], \end{array}$$ (1)

where

$$\begin{array}{c} \theta_{i}=tan\left(\frac{\vartheta_{i}-\psi_{i-1}}{2}\right), \end{array}$$ (2)

and *ψ*_*i*−1_ is the direction of the external attractive field at the position (*x*, *y*, *z*)_*i*−1_. The transformation in Eq 2 sets the domain of the model to (−∞, ∞), allowing a direct implementation of the Normal distribution. By providing a closed-form expression, this model marginalization (or renormalization) enables the comparison of models generated with data of different resolutions and using different simulation scales. Furthermore, it avoids using the Bessel functions for the normalization of the usual von Mises distribution for the angular domain. With this definition, the variable *θ*_*i*_ is locally defined in reference to *ψ*. Furthermore, *α* weights the difference between the future and current direction, and *β* the difference between the *i*^*th*^ step vector direction and the direction of the attractive force. Thus, high values of *α* favor a straight axonal trajectory while *α* → 0 results in a very tortuous one (S1 Fig). Similarly, if the external attraction is very high (*β* → ∞), axons tend to align to the external field gradient lines, while if this attraction is low (*β* → 0) they do not follow any preferential direction (S1 Fig). The same model in Eqs 1 and 2 is applied to the elevation angle *φ*_*i*_, to define *P*(*ϕ*_*i*_|*ϕ*_*i*−1_), with $\phi_{i}=tan\left(\frac{\varphi_{i}-\psi_{0,i-1}}{2}\right)$.

Notably, the model can be equivalently written as follows:

$$\begin{array}{c} \theta_{i}=\frac{\alpha }{\alpha +\beta }\theta_{i-1}+\xi_{i}, \end{array}$$ (3)

where

$$\begin{array}{c} \xi_{i}∼N\left(0,\frac{1}{2(\alpha +\beta )}\right). \end{array}$$ (4)

With this formulation, it becomes clear that at each step of the chain *θ*_*i*_ conditioned to the step before, *θ*_*i*−1_, follows a Normal distribution with mean $\mu =\frac{\alpha }{\alpha +\beta }\theta_{i-1}$ and variance $\sigma^{2}=\frac{1}{2(\alpha +\beta )}$ (Fig 1A).

A major advantage of our model is that the main parameters *α* and *β* can be directly estimated from real axonal trajectories extracted from data. Finally, as mentioned before, our model can be rescaled in function of data spatial sampling, allowing closed forms to obtain equivalent parameters in smaller or higher scales (see Model renormalization in Supporting Information).

#### Branch formation

The algorithmic implementation of branch occurrence is described in Fig 1B. After each time unit *t*_*j*_, if a certain branching “condition” is fulfilled, then a branching point is placed at the axon tip or at any other step performed during *t*_*j*_. If “condition” is false, *t*_*j*+1_ begins and the axon continues growing (not shown). “condition” can be arbitrarily set by the modeler and, for example, follows a uniform probability law. Alternatively, it can be estimated from real data. In the first case, “condition” is fulfilled if a random number from 0 to 1 is smaller or equal to the branching probability *P*_*b*_. In this case, the branch is placed randomly at one of the steps performed during *t*_*j*_. Branches are born with random initial angles drawn from the distribution *ω*. In this model, we also considered branch density (λ_*b*_), such that a new branch appears if its distance to the previous branch is higher or equal to a random Poisson number with parameter λ_*b*_. Finally, the parameter *b*_*l*_ determines the maximum order of branches (*i*.*e*. *b*_*l*_ = 0 means no branching, *b*_*l*_ = 1 only first order branches, etc.). *P*_*b*_, *ω*, λ_*b*_ and *b*_*l*_ can be estimated from data or imposed (Table 1).

#### Growth stopping conditions

By default in our framework, axons stop growing when one of their tips reaches a predefined region. This region depends on the axon spatial environment -when defined- or can be set arbitrarily (*X*_*max*_, see Table 1). It is also possible to indicate a pre-defined maximum length for branches, following for example a Normal law.

#### Generation of a variety of axonal morphologies

**Fig 2:**
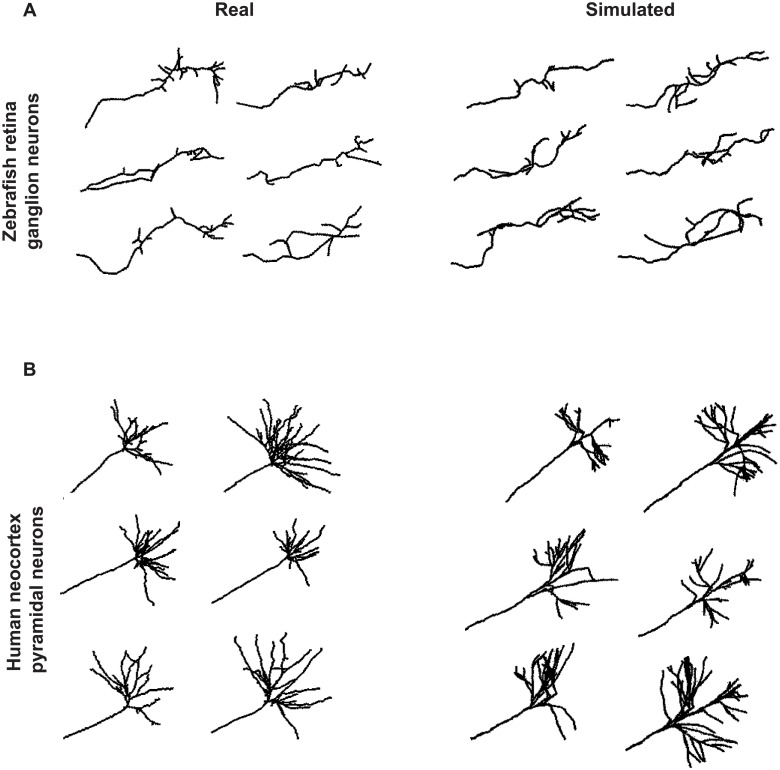
Examples of axonal morphologies generated by the model and comparison to real data. (A) Real (left) and simulated (right) axonal trees of zebrafish retina ganglion neurons. (B) Real (left) and simulated (right) axonal trees of human neocortex pyramidal neurons. Real axonal trees were obtained from the Neuromorpho.org database. Simulated axons where generated in isolation, using the following parameter values (Zebrafish/Human): for the main axon ***α*** = 15/10 and ***β*** = 2/30; for the branches ***α*** = 20/100 and ***β*** = 0.5/10, **Δ*ρ*** = 1/3, ***ψ*** = 0 rad ∀*x* / 0 rad. for *x* ≤ 60 *μ*m, +(−)1.3 rad for *x* > 60 *μ*m and *y* > 0(*y* ≤ 0), ***P***_***b***_ = 0.5/0 for *x* < 60 *μ*m and 0.3 otherwise, ***ω***: uniform in all the space/ uniform in a solid angle of $\frac{\pi }{4}$ respect to the neurite from which the branch emerges, **λ**_***b***_ = 6/1, ***b***_***l***_ = 2/2, ***n***_***max***_ = 10/1, ***X***_***max***_ = 80/110. In addition, for the human neocortex pyramidal neurons, branch maximum lengths follow a normal distribution of mean 100 *μ*m and standard deviation 70 *μ*m.

As illustrated in Fig 2, our framework can generate axonal trees with realistic and heterogenous morphologies. Indeed, the arborization patterns of both zebrafish retina ganglion neurons (left) or human cortical pyramid neurons (right) were successfully reproduced upon simulation of single axons in isolation (listed in the NeuroMorpho.Org database [29–31]). In these examples, the parameters were set manually: we first varied simultaneously *α*, *β* and Δ*ρ* to match the axon sinuosity, and then set the parameters related to branch occurrence to simulate the arborization pattern specific to each population.

### Mechanical interactions between simultaneously growing axons

To mimic the crowded environment of the brain, we implemented *via* volume exclusion the mechanical and spatial constraints imposed by surrounding tissues an cells, which can occur upon interaction with another axon, or with the edges of the cavity *ξ* in which axons grow. We considered two possibilities for each artificial time point. If space permitted, axon tips grow with a maximal speed of $v_{max}=\frac{n_{max}\Delta \rho }{\Delta t}$ during *t*_*j*_. If, on the contrary, axon tips encounter another axon, or the geometrical limits of *ξ* before accomplishing *n*_*max*_ steps, they will retract the last few (*n*_*r*_) steps realized during *t*_*j*_ (Fig 1C). Then, they will try to regrow in another direction until *n*_*max*_ steps are accomplished (Fig 1C, CASE A). However, if a second obstacle is encountered within the same time frame (Fig 1C, CASE B), axon tips will stop their growth after the retraction of the last *n*_*r*_ steps, and will try again growing only in the next time point (*t*_*j*+1_). Such an alternance of growth and repulsion steps during the axonal elongation process has been described in the literature [17, 23, 25]. Gallo and colleagues ([7]), for example, observed that neurons growing under mechanical constraints sample repeatedly the obstacles they encountered, until they find a free way. Furthermore, Schier and colleagues ([32]) mentioned the existence of inter-neuron contacts during development, proposing the so-called growth and repulsion mechanism. For the sake of computational time, as well as to mimic real timing constraints imposed by the developmental program, each axon tip has a limited global number of trials before it stops growing (counter). The maximum value of trials upon interaction (counter_max_) should be estimated or fixed. The axonal diameter *d* may be measured from data or adapted from already published values. Finally, the shape and dimensions of the growth cavity *ξ* should be adapted to the case under study (see Table 1).

In order to handle volume exclusion, the growth of each axon is simulated sequentially in each time point. After each step, the algorithm checks that the new position of the axon tip is at least one diameter (*d*) away from every other axonal structure or cavity limits (*ξ*). If this condition gets false (mechanical obstacle), the algorithm proceeds as described in the previous paragraph.

#### Simulations of collective axon growth

**Fig 3:**
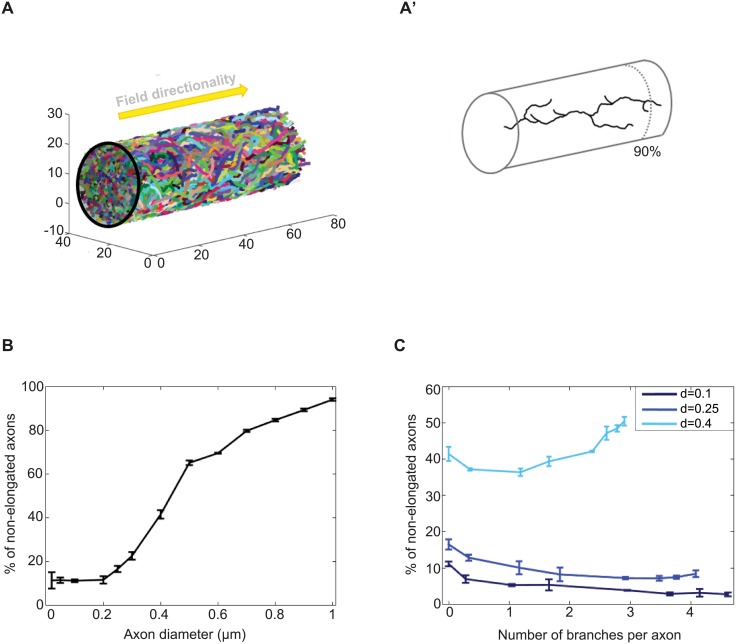
Simulations of axons growing collectively in a confined environment. (A) 400 axons were simulated growing inside a cylinder of fixed diameter and length. Left: whole population. Right: example of an individual properly-elongated axon generated with ***d*** = 0.4 *μ*m and ***P***_***b***_ = 0.5. Axons were defined as properly elongated if at least one of their branch tip reached 90% of the cylinder length. The external field (*ψ*) directionality is represented as an arrow. (B) Percentage of non-elongated axons in function of the axon diameter value. (C) Percentage of non-elongated axons in function of branch number for different axonal diameter values. The error bars in B and C represent the standard deviation observed after running 3 simulations. The following parameter values were used: ***α*** = 9 and ***β*** = 2, **Δ*ρ*** = 1, ***ψ*** = 0 rad, ***ω***: uniform in all the space, **λ**_***b***_ = 15, ***b***_***l***_ = 1, ***d***: indicated in each case, ***Num***_***ax***_ = 400, ***n***_***max***_ = 6, ***n***_***r***_ = 2, **counter**_**max**_ = 140, ***X***_***max***_ = 70 and ***ξ***: tube of radius 13 *μ*m. ***P***_***b***_ = 0 was used in B and for the “no branch” condition in C. Increasing values of ***P***_***b***_ were used to increase the number of branches per axon (S2B Fig).

Fig 3 illustrates the importance of taking into account spatial competition during axonal growth. 400 axons were considered growing in a cylinder of fixed diameter and length (Fig 3A). We first analyzed the impact of considering increasing axonal diameter in non-branching axons, and defined the percentage of non-elongated axons as the proportion of the population that did not reach the extremity of the cylinder (90% of the tube). As shown in Fig 3B, this proportion grows in a logistic way with the diameter size, due to the decreasing fraction of free volume and increasing spatial competition between axons. A similar trend is observed when increasing axon number (*Num*_*ax*_) (S2A Fig). For a fixed diameter (*d* = 0.1, *d* = 0.25 or *d* = 0.4*μ*m) and a fixed number of axons (*Num*_*ax*_ = 400), we then analyzed the percentage of non-elongated axons when increasing the probability of branching (*P*_*b*_), and thus the number of branches per axon (Fig 3C and S2B Fig). For axonal diameters of 0.1 and 0.25 *μ*m, forming branches decreased the percentage of non-elongated axons for any branching probability, reflecting a higher chance to have one axon branch tip reaching the extremity of the cylinder. For *d* = 0.4 *μ*m, forming more than one branch was prejudicial to the overall growth success of the population, reflecting an increase in neuronal density and in the probability of encountering mechanical obstacles. In contrast, increasing counter_max_ (duration of growth) promotes axon elongation (S2C Fig), enabling axons to complete their growth within the considered time window.

### Biological model: *Drosophila* Mushroom Body *γ* neurons

**Fig 4:**
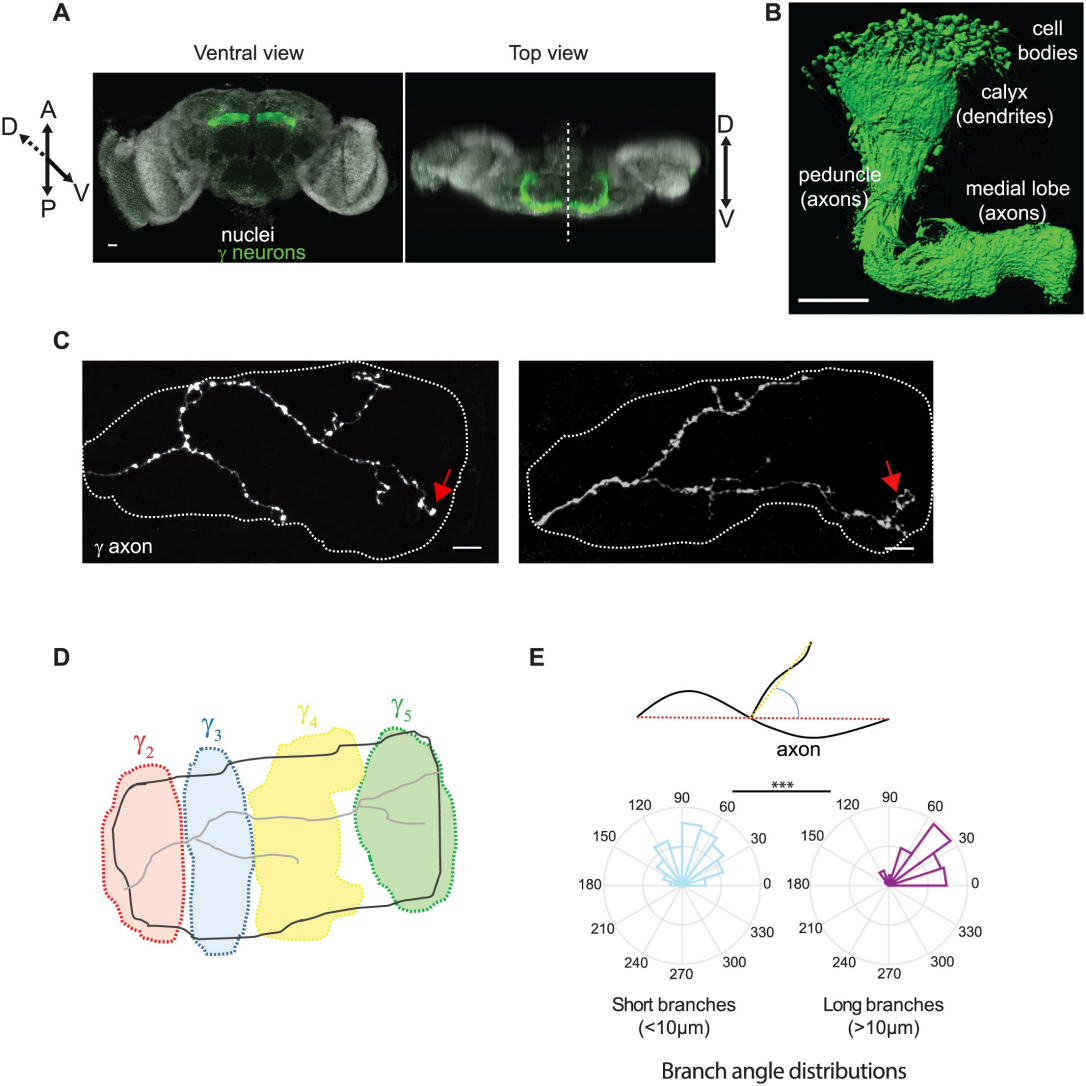
Characteristics of adult Mushroom Body *γ* neurons. (A) Wild-type adult *Drosophila* brain expressing the membrane-tagged CD8-GFP construct in *γ* neurons, under the control of the MB009B-Gal4. Nuclei are labeled in white with DAPI. The dotted line on the top view image corresponds to the midline. (B) 3D reconstruction of one Mushroom Body where *γ* neurons only were labeled. Genotype: MB009B-Gal4/UAS CD8-GFP. (C) Axonal arborizations of two individual adult *γ* neurons labeled by GFP using the MARCM technique. Confocal images taken along the z axis were projected (Maximum Intensity Projection). Dotted lines delimit the shape of the medial lobe. Red arrows indicate axon tips that reached the extremity of the medial lobe. (D) Schematic representation of the different anatomical domains (distinguished with different colors) defined along the medial lobe and innervated by distinct input and output neurons. Adapted from [34]. Scale bars: 20 *μ*m in A, 50 *μ*m in B and 10 *μ*m in C. (E) Top panel: schematic representation of how directionality angles were measured. We have considered the angle between the vector defined by the first and last point of the mother branch (red segment), and the vector defined by the first and last point of the considered branch (yellow segment) projected on the xy plane. Bottom panel: frequency distribution of the directionality angles of long (>10 *μ*m, type I) and short (<10 *μ*m, type II) branches measured on reconstructed wild-type adult *γ* axons. The frequency values are presented as a percentage of each group (n = 97 and 283 for type I and II branches respectively). The middle circle represents 10 and 15% for type II and I respectively. *** stands for *p* = 4.4^−13^ (Kruskal Wallis test).

To understand the importance of considering mechanical interactions and confinement when studying axons growing as a population *in* *vivo* in brains, we applied our model to Mushroom Body (MB) *γ* neurons. These neurons project stereotypically in each hemisphere of the *Drosophila* brain (Fig 4A). MB *γ* axons (about 650 in total) fasciculate proximally to form a dense fiber projecting ventrally: the peduncle (Fig 4B). More distally, adult axons de-fasciculate to innervate the so-called medial lobe where they form branches of various lengths, with at least one reaching the distal tip of the medial lobe (Fig 4C, red arrows and [33]). Remarkably, a range of arborization patterns is typically observed within populations of genetically identical *γ* neurons. As described previously by Rubin and colleagues [34], MB *γ* axons establish contact with specific sets of input and output neurons projecting to functional and anatomical compartments distributed along the medial lobe (Fig 4D).

To quantitatively analyze the morphology of individual axons within the population, we genetically labeled single MB *γ* neurons and imaged their adult arborization patterns by confocal microscopy. We then reconstructed the skeletons of each individual axonal trees (n = 43; available at NeuroMorpho.org), and included them all in the same reference lobe after normalization (Supporting information and S3A–S3D Fig). As shown in S3C and S3D Fig, reconstructed axons spread over the entire reference lobe and exhibited a wide range of morphologies, indicating that our database is representative of the variety of growth patterns that *γ* axons may adopt. Analyzing the average directionality of individual axonal segments along the medial lobe revealed that MB *γ* axons are strongly oriented toward the midline (S3E Fig). Furthermore, measuring the deviation angles of branches from the branch they emerge, revealed that branches could be classified into two groups with distinct properties. While long (>10 *μ*m) branches were mainly oriented relatively parallel to the lobe axis (Fig 4E), sometimes reaching the distal end of the medial lobe (Fig 4C), short branches (<10 *μ*m) exhibited no bias in directionality (Fig 4E). These observations suggested that long and short branches (that we named type I and type II branches respectively) may be generated *via* distinct mechanisms.

To better understand how branches are generated and thus implement them in our framework, we imaged in real-time maturing brains expressing GFP in single *γ* neurons. MB *γ* neurons are born during embryogenesis and early larval stages but then undergo developmental remodeling [34–38], such that growth and branching of adult axonal trees occur during metamorphosis. Although live-imaging at this stage did not allow us to follow the entire growth of *γ* axons, it enabled us to dynamically record parts of this process. Consistent with our analysis of fixed samples, two main types of branches were observed in movies: very dynamic short branches (type II, asterisks), and more static longer branches (type I, arrows) (S1 Video and S4A Fig). Type II branches exhibited series of growth and retraction events and typically measured between 2 and 10 *μ*m (80% between 2 and 5 *μ*m and 18% between 5 and 10 *μ*m, n = 484, see S4B Fig). Longer type I branches had a dynamic activity restricted to branch tips, and were on average longer than 10 *μ*m. We thus included both types of branches in our model, such that two scenarios can occur at the end of each *t*_*j*_. In the first one, the axon generates a type I branch that will then elongate following the previously described rules in Fig 1. In the second one, no type I branch is generated. A type II branch is then formed if its distance to the previous branch is higher or equal to a random number from the Poisson distribution with parameter λ_*b*_. This branch will appear and disappear with different uniform random angles and get stabilized if it establishes a contact with another branch. Its length is drawn from the distribution measured from data and described previously. As type II branches are quite short and dynamics, we did not consider their volume in our model, but only their formation and stabilization.

### Parameter estimation

Beyond enabling quantitative description of axonal trees, reconstructions of real axons allowed us to directly estimate, or calibrate, all the morphological and spatial parameters from individual axons grown *in* *vivo* as a population (see values in Table 2). Temporal parameters were arbitrarily fixed and invariant for all the experiments.

**Table 2:** Parameter values and estimation procedure.

Parameter	Value	Method
**Morphological**		
*α*	7.45	Estimated from data
*β*	1.67	Estimated from data
Δ*ρ*	1 *μ*m	Estimated from data
*ψ*	Field described in S6B Fig	Estimated from data
*P*_*b*_	0.15	Estimated from data
*ω*	Random uniform	Hypothesis
λ_*b*_	6.2	Estimated from data
*b*_*l*_	1 (type I) 2 (type II)	Observed from data
*d*	0.23 *μ*m	Calibrated from data and bibliography
*Num*_*ax*_	650	From bibliography
**Dynamic**		
*n*_*max*_	6	Fixed
*n*_*r*_	2	Fixed
counter_max_	140	Fixed
**Spatial**		
*X*_*max*_	70 *μ*m	Estimated from data
*ξ*	See S6D Fig	Estimated from data and bibliography

***α* and *β***: To estimate these parameters, we considered that each *k*^*th*^ reconstructed axon of length *M* can be represented by the sequence $\theta_{i}^{k}$, applying Eq 2 to each step of length Δ*ρ*. It can be shown that the variance of $\theta_{i}^{k}$ is

$$\begin{array}{c} \sigma_{\theta_{i}^{k}}^{2}=\sigma_{0}^{2}\sum_{i=1}^{M-1}\gamma^{2i}=\sigma_{0}^{2}\frac{1-\gamma^{2M}}{1-\gamma^{2}} \end{array}$$ (5)

where

$$\begin{array}{c} \gamma =\frac{\alpha }{\alpha +\beta };\sigma_{0}^{2}=\frac{1}{2(\alpha +\beta )}. \end{array}$$ (6)

When *i* → ∞ we obtain the expression

$$\begin{array}{c} \sigma_{\theta_{\infty }^{k}}^{2}=\frac{\sigma_{0}^{2}}{1-\gamma^{2}}. \end{array}$$ (7)

It can also be shown (Supporting information) that the variance of the difference $\theta_{i}^{k}-\theta_{i-1}^{k}$ (for *i* → ∞) is

$$\begin{array}{c} \sigma_{\Delta \theta_{\infty }^{k}}^{2}=\frac{2\sigma_{0}^{2}\left(1-\gamma \right)}{1-\gamma^{2}}. \end{array}$$ (8)

To obtain the estimations ${\hat{\alpha }}_{k}$ and ${\hat{\beta }}_{k}$, we assume that the axons are long enough, and calculate $\hat{\sigma_{\theta_{\infty }^{k}}^{2}}$ and $\hat{\sigma_{\Delta \theta_{\infty }^{k}}^{2}}$, from where we get $\hat{\gamma }$ and $\hat{\sigma_{0}^{2}}$ to finally apply Eq (9)

$$\begin{array}{c} {\hat{\alpha }}_{k}=\frac{\hat{\gamma }}{2\hat{\sigma_{0}^{2}}},{\hat{\beta }}_{k}=\frac{1}{2\hat{\sigma_{0}^{2}}}-{\hat{\alpha }}_{k}. \end{array}$$ (9)

and obtain the estimates of the model parameters for each neuron *k*. To estimate the parameters of a population containing *K* axons, we consider the distributions of parameters (${\hat{\alpha }}_{k},{\hat{\beta }}_{k}$) obtained from individual axons using Eq 9. However, the distributions of the estimated values were not well described by their mean or median. Thus, we chose the couple of values ($\hat{\alpha },\hat{\beta }$) that maximized the similarity between the distribution of parameters estimated from data and that obtained from simulations with different values of (*α*, *β*), taking into account axon-axon interactions (S4 Fig and Supporting information). The estimated values were: $\hat{\alpha }=7.45$ and $\hat{\beta }=1.67$ (Table 2).

**Δ*ρ***: was set to 1 *μ*m, to be consistent with the general axonal diameter in the images, and avoid oversampling (see Supporting Information and Table 2).

***ψ***: the external attractive field was estimated based on the observed directionality of real axons, as neither the identity nor the source of the cue(s) guiding the growth of *γ* axons are currently known. We placed the attractive source at the end of the medial lobe, and evaluated different gradient geometries by comparing the similarity between real axon orientation and field directionality (S6A–S6C Fig and Supporting information). The source configuration maximizing this similarity was selected for further analysis, see Table 2.

***d*** and ***Num***_***ax***_: the axonal diameter was estimated from published electron microscopy images [39], and optimized by simulations (see S6E Fig and Supporting information). The selected diameter value corresponds to the first elbow of the obtained logistic function (*d* = 0.23 *μ*m). *N*_*max*_ = 650 was obtained from the work of [34] (Table 2).

***ξ*** and ***X***_***max***_: *in vivo*, adult *γ* axons grow as a population, in a confined environment defined by surrounding neuronal and glial cells (Fig 4A and [34]). To consider mechanical constraints that underlie axon growth in the crowded environment of a maturing brain, we imposed a spatially-restricted environment mimicking MB lobe geometry, and defined based on our confocal images as well as on the work of Aso et al. [34] (Supporting information and S6E Fig).

***P***_***b***_, ***ω***, **λ**_***b***_ and ***b***_***l***_: We first estimated the probability *P*_*b*_ as the mean of main axon lengths (93 *μ*m) divided by *n*_*max*_ to obtain -roughly- the number of time points (*t*_*j*_) it takes to grow. By simply dividing the mean number of type I branches per axon (2.25) by this number, we obtained *P*_*b*_ = 0.15. Based on our observations of real samples, we then hypothesized that branches (types I and II) are born initially with uniform random angles (*ω*), and that type I branches can create type II branches but not type I (*i*.*e*. *b*_*l*_ = 1 for type I branches and 2 for type II (Table 2)).

### Impact of branching on the growth of *γ* axon population

**Fig 5:**
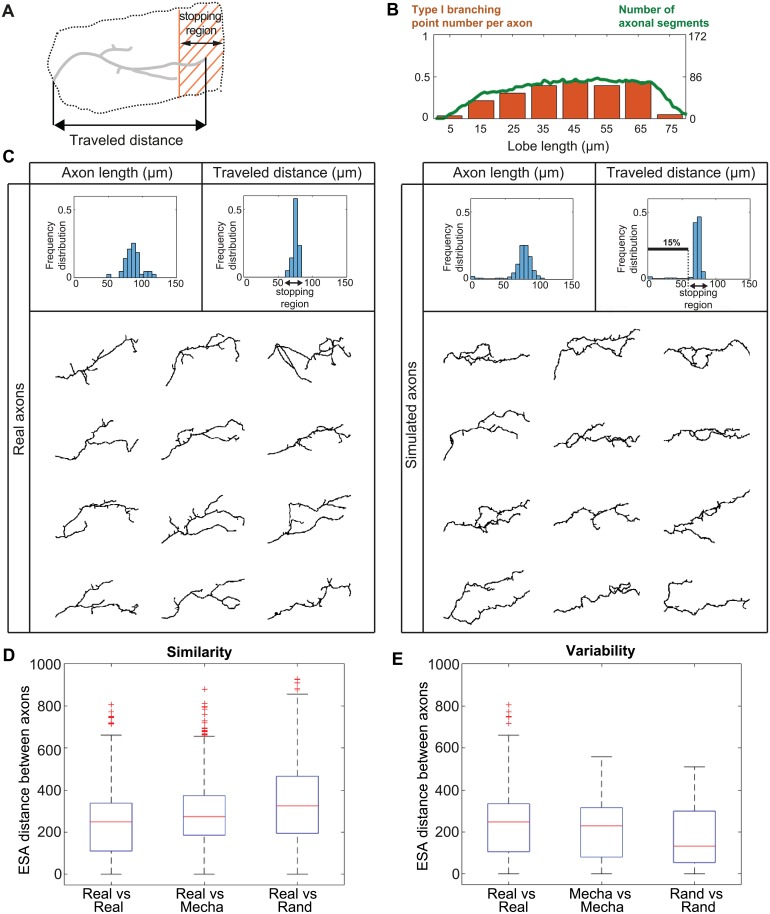
Simulation of *γ* axon growth within a population of interacting axons. (A) Schematic representation of a single main *γ* axon within the medial lobe. Both the stopping region (shaded region) and the traveled distance (projection along the horizontal axis) are indicated. Axonal trees that fail to elongate a branch reaching the stopping region are considered as non-elongated. (B) Correlation between the spatial distribution of type I branching point number and axonal density. The orange bars represent the number of type I branching points per axon along the lobe axis. The green curve represents the number of axonal segments found in each lobe region. Data from reconstructed real axons were used for this analysis. (C) Top: Frequency distributions of 2D-projected main axon lengths (left panels), and traveled distances within the medial lobe (right panels). Data from real wild-type *γ* axons are shown in the left panels (n = 43), and data from simulated ones in the right panels (n = 650). The black arrows indicate the stopping region. The distributions of 3D main axon lengths are shown in S8A Fig. The definition of main axon is provided in Supporting information. Bottom: Examples of reconstructed real single wild-type *γ* axons (left) and simulated *γ* axons (right). (D) Measure of similarity between real and simulated axons. Boxplots of inter-axon distances within the population of real axons (real, n = 43), and between real and simulated axons, considering either mechanical (mecha) or random (rand) branching (n = 50 in both cases). (E) Measure of intra-population morphological variability. Boxplots of inter-axon distances within the population of real axons (real, n = 43), and within the population of simulated axons, considering either mechanical or random branching (n = 50 in both cases). Inter-axon distances were calculated with the ESA distance [40]. Boxplots were created following the original plotting convention of Tukey. The central mark indicates the median, the bottom and top edges of the box indicate the 25^*th*^ and 75^*th*^ percentiles, respectively. The whiskers extend to the most extreme data points not considered as outliers. Outliers are plotted individually using the ‘+’ symbol.

Using our model and the parameters estimated from data, we simulated entire populations of *γ* axons. To estimate the capacity of axons to successfully grow, and extend until the extremity of the medial lobe, we defined a “stopping region” of about 20 *μ*m-wide at the end of the medial lobe (Fig 5A). Although real axons do not all sharply stop at the extremity of the medial lobe (midline), they all reach this region. We thus considered as non-elongated axons those that did not reach this region. In this condition, about 10% of simulated *γ* axons (n = 3 simulations) failed to elongate properly.

To assess the validity of our branch occurrence hypothesis (random uniform), we then analyzed the distribution of normalized type I branching point numbers along real lobe axes (Fig 5B, orange bars). This revealed that, *in* *vivo*, type I branching points are not uniformly distributed throughout the lobe, but rather peak in the most central part. Such a distribution correlates with that of axon density (or occupation rate) along the medial lobe (Fig 5B, green line). This observation led us to consider an alternative hypothesis in which branch occurrence may be favored in regions of high density and increased spatial competition.

Thus, we proposed that branching condition is true when the axon tip encounters mechanical obstacles (branching upon contact) during a given time interval (CASE B in Fig 1C). Considering such a mechanical branching, we performed new simulations of entire *γ* axon populations, and observed a reduction of the percentage of non-elongated axons (4.9 ± 0.22%; n = 3 simulations). To more precisely assess the similarity between real and simulated axons in this condition, we compared the distributions of main axon lengths (defined in Supporting information), as well as the distributions of distances traveled within the medial lobe (defined by the distances from the lobe entry point to the end point projected along the medial lobe axis, scheme in Fig 5A). As shown in Fig 5C, the distribution profiles of real and simulated axons were very similar.

In term of branching, simulated *γ* axons had on average 2.0 ± 0.01 type I branches (n = 3 simulations; standard deviation *σ* = 1.68), a number very close to that of real axons (2.25 with a standard deviation of 1.1). Furthermore, they exhibited morphologies matching those of real axons (Fig 5C).

To quantify the overall similarity between simulated and real axonal trees, we used the distance between trees developed by [40]. This measurement takes into account the length, the morphology, and the directionality of main axons, as well as branching characteristics. As shown in Fig 5D and S7B Fig, the distribution of distances between all pairs of real and simulated axons is close to that of distances between all pairs of real axons and, remarkably, is significantly closer to that obtained with axons simulated using the random branching hypothesis (p value between random and mecha of 3.3*e*^−15^). To then determine if our model accurately reproduces intra-population variability, we compared the distances between each couple of real axons to those between each couple of simulated ones (Fig 5E and S7C Fig). The distributions for real axons and axons simulated with mechanical branching were close, while that for axons simulated with random branching was significantly different (p value between random and mecha of 9.6*e*^−7^). This analysis highlighted that our model recreated morphological features that were not initially imposed by the model, but rather emerged from its rules.

Together, integrating axon-axon interactions in our model generated populations of *γ* axons with a realistic range of morphologies. Furthermore, this revealed that branching in response to physical constraints increases the chance that axons successfully elongate, and reproduces the intra-population variability of morphologies observed for real axons.

### Out-competition of non-branching axons by branching ones: Interpretation of mutant phenotypes

**Fig 6:**
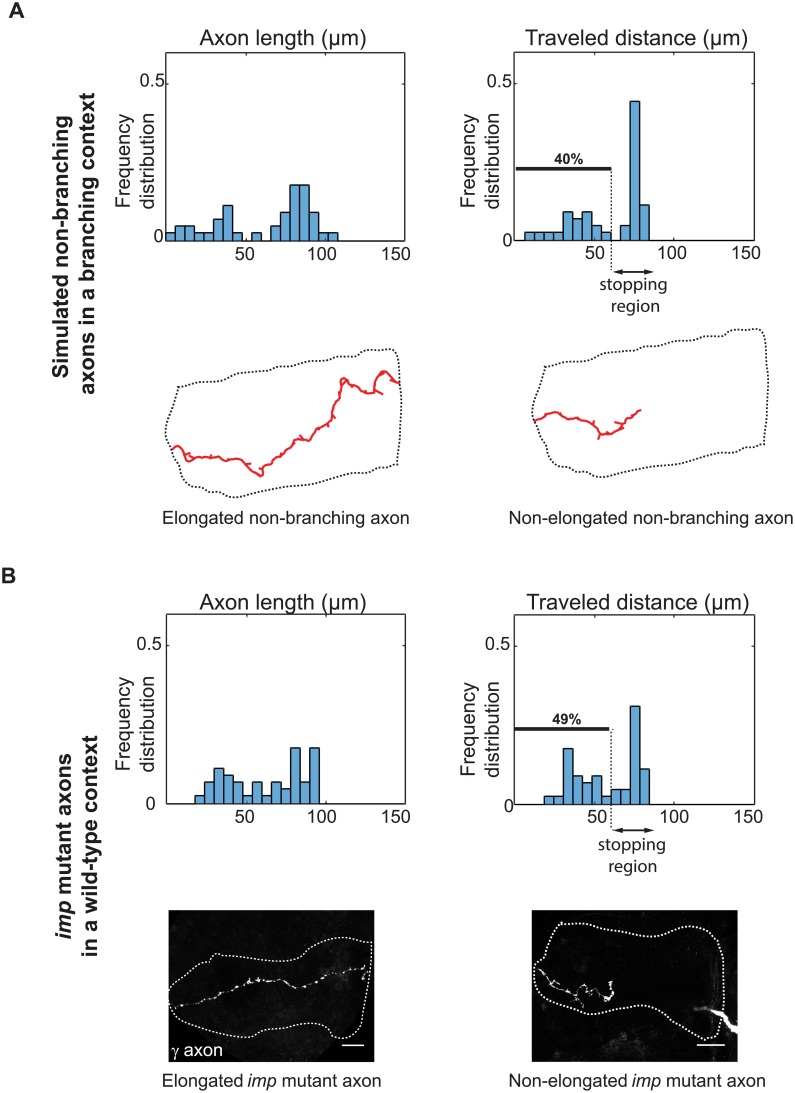
Out-competition of non-branching axons by branching ones. (A) Top: frequency distributions of 2D-projected axon lengths and traveled distances of single simulated non-branching axons grown in the context of otherwise branching axons. 40% of axons fail to reach the lobe extremity in this condition (n = 45). Bottom: examples of fully elongated (left) and growth-defective (right) simulated single axons. (B) Top: frequency distributions of axon lengths and traveled distances of single *imp*^7^ mutant axons grown in the context of a wild-type population. A bimodal behavior similar to that generated by simulations is observed (n = 45). Lower panels: example of fully elongated (left) and growth-defective (right) *imp*^7^ single axons grown in a wild-type environment and labeled with GFP. Scale bar 10 *μ*m.

So far, we have considered in our simulations that the whole population of axons follows the same rules, and have analyzed the emergent collective phenomena. We next wondered what would happen if the properties of only a single neuron would be altered in a context where the rest of the population grew according to our model. To address this question, we performed 45 independent simulations where a single axon unable to generate type I branches grew among a population of surrounding axons capable of branching. As shown by the distributions of axon lengths and traveled distances of single non-branching neurons, a bimodal behavior emerged with about half of the simulated single axons failing to grow properly (Fig 6A). This proportion is much higher than that observed in condition where all axons in the population do not form branches (10% in this condition).

The bimodal axon growth distribution we observed for non-branching single neurons was reminiscent of the growth pattern described for single neurons mutant for the *imp* gene [41, 42]. To quantitatively compare distributions, we took advantage of a collection of 45 confocal images obtained from *Drosophila* brains in which axons mutant for *imp* grew in the context of an otherwise wild-type population. These neurons, labeled with GFP, were reconstructed, and the distribution of their axon length and traveled distance plotted. Strikingly, as shown in Fig 6B, the bimodal distribution profiles of real *imp* mutant axons were very similar to those displayed in Fig 6A, with about half of the mutant individual axons failing to reach the extremity of the medial lobe. Furthermore, the morphology of reconstructed *imp* mutant axons was very similar to that of individual simulated axons (S8B and S8C Fig). In both cases, indeed, few short side branches were observed along the main axon, and a mixture of short and long axons was observed (compare Fig 6A and 6B, lower panels and see [41]). These results thus suggest that branching deficiency might be the primary defect induced by *imp* inactivation, resulting in axon growth defects exacerbated by a competition with surrounding wild-type neurons. They also demonstrate the biological relevance of our mathematical framework, and its capacity to propose a mechanistic interpretations of *in* *vivo* phenotypes. An interesting prediction of our model is that the defective growth of individual non-branching axons can be rescued by increasing their apparent growth speed. Indeed, increasing *n*_*max*_ in non-branching axons surrounded by branching axons increased their chance to properly elongate, both in the context of a theoretical cylindric volume (S9A Fig), and in the context of an *in* *vivo* environment (increasing *n*_*max*_ by five fold reduces the percentage of non-elongating non-branching axons to 5%; not shown). In contrast, increasing *n*_*max*_ did not significantly affect elongation success in a homogeneous population of growing axons (S9B Fig).

### Model sensitivity to *α*/*β* values

Our model of individual axon growth relies on two main parameters estimated from *in vivo* data: *α* and *β*. To investigate how differences in parameter values affect the system, we separated the wild type *in vivo* data set in two random halves, and estimated the parameters separately in each population. Similar values of *α* and *β* were obtained for the two populations (S5B” Fig). We then simulated the growth of *γ* axon populations using these two pairs of parameters, and obtained a growth efficiency of above 95% in each case (data not shown). This reveals the stability of the model in response to small changes in the parameter values, as well as the parameter estimation coherence within the data set.

**Fig 7:**
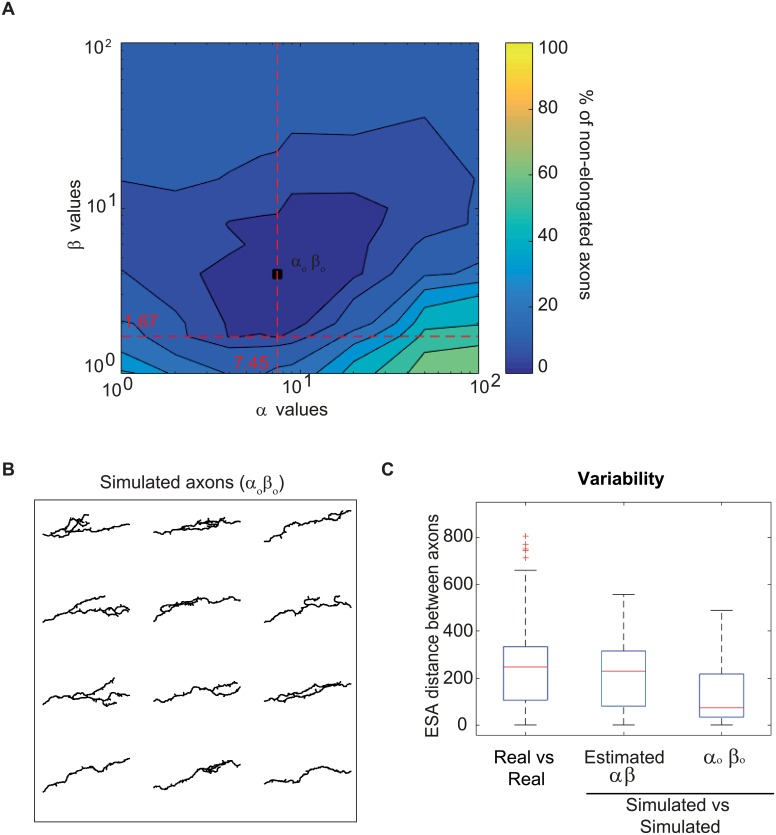
Impact of *α* and *β* values on axon morphologies. (A) Percentage of axons failing to reach the lobe extremity in function of pairs of parameter values. The dotted lines correspond to the parameter values estimated from data (see Parameter Estimation). The square shows the pair of parameter values minimizing the percentage of axons that do not reach the lobe extremity (*α*_*o*_ and *β*_*o*_). (B) Examples of *γ* axon morphologies from simulations with the parameters that optimize axon growth at the population level (*α*_*o*_ *β*_*o*_). (C) Boxplots of inter-axon distances within the population of real axons (real, n = 43), axons simulated with the estimated parameters (estimated *α* *β*, n = 50), and axons simulated with the *α*_*o*_ *β*_*o*_ parameters (n = 50). Inter-axon distances were calculated with the ESA distance [40]. Boxplots were created following the original plotting convention of Tukey. The central mark indicates the median, the bottom and top edges of the box indicate the 25^*th*^ and 75^*th*^ percentiles, respectively. The whiskers extend to the most extreme data points not considered as outliers. Outliers are plotted individually using the ‘+’ symbol.

To further test the robustness of the model, we then calculated the percentage of the *γ* axon population that fail to elongate in simulations of collective growth upon larger variations in the *α* and *β* values (see resulting map in Fig 7A). Remarkably, the combination of parameter values estimated from data, and used in our model (*α* = 7.45 and *β* = 1.67) is close to the theoretical combination that minimizes the percentage of axons showing defective growth (*α*_*o*_ *β*_*o*_) (Fig 7A).

Interestingly, we noticed that the *α*_*o*_ *β*_*o*_ combination, while optimal for axon growth efficiency, generated axons with a reduced complexity (Fig 7B; to be compared with Fig 5C), and a lower number of type I branches (1.6 +/- 1.4 *vs* 2.25 +/- 1.1 for real axons). As further illustrated by the measure of intra-population morphological variability (Fig 7C), the population of axons simulated with these values was more homogeneous than the real one, failing to reproduce the diversity of axon morphologies observed *in* *vivo* (p value between axons simulated with estimated parameters and *α*_*o*_ *β*_*o*_ of 1.5*e*^−44^). Thus, the pair of parameters estimated from real data does not only represent an optimal combination ensuring efficient growth, but also generate biologically-relevant morphological diversity and complexity.

## Discussion

In this work, we have proposed a stochastic model for 3D axon growth and branching that takes into account spatial constraints imposed by the environment, as well as physical interactions between neighboring axons of the growing population. As shown, the proposed model is not only generative of axonal morphologies, but also explicative and predictive.

### Model parameters reflect biological properties

A major strength of this model is that it relies on parameters that are related to the biological process of axon growth, and can be estimated or calibrated from data or literature (when available).

Individual axon path, for example, is modeled by a succession of discrete steps, governed by a Gaussian Markov Chain with two parameters that are linked to the physiological and mechanical properties of individual axons. Specifically, it embeds a first term reflecting the axonal rigidity (*α*), and a second term modeling the attraction provided by an external field (*β*). Thus, the model includes three main influences on axon growth: rigidity, attraction towards a target area and randomness (representing not only the inherent stochasticity of the biological process, but also the presence of other surrounding cells). Similar ways of modeling axonal growth have already been described in previous work [43–47], considering different mathematical formulations. Most of previous models, however, were developed in 2D, and did not allow the estimation of parameters from data. Furthermore, they relied on many parameters, and sometimes parameters without direct biological meaning. Our 3D model overcomes these limits, in part because it is invariant to data spatial sampling, which is an important advantage when dealing with discrete models and discrete datasets of possibly different spatial resolutions. Classically, the parameters of such models can be estimated from data with second order statistics. However, we observed that the proposed model is broken when axons growing in a population context hit surrounding cells or other axons. Therefore, we proposed a scheme that alternates population simulation and parameter estimation to take into account these environmental constraints, and accurately estimate the parameter values. Remarkably, the parameters we thus estimated from real neurons generated populations of axons that not only recapitulated, but also predicted the properties of real axons growing *in* *vivo* in the context of the *Drosophila* brain.

Model parameters can be divided into three articulated sets (morphological, spatial and temporal), which enables both flexible and step-specific dissections of the axon growth process, and the study of a wide-range of growth patterns. Highlighting the versatility of our model, a variety of morphologies could be simulated by adapting parameter values (Fig 2).

### Origin of morphological diversity within a population of genetically identical neurons

Another major strength of our model is that it can provide mechanistic interpretations of neuron behavior. For example, the diversity in *γ* axonal trees observed *in vivo* is well reproduced and explained by our model that generates collections of neurons with unique morphology. Part of the variability observed after simulation is triggered by intrinsic factors, including the intrinsic stochasticity of the axon growth process modulated by the properties of the axons. Increasing axon sensitivity to the attractive field, indeed, generated populations of neurons with reduced morphological heterogeneity (Fig 7B and 7C), suggesting that differences in intrinsic properties may partly explain the various degrees of morphological variance observed *in* *vivo* in different populations. Interactions with the surrounding environment also largely contribute to the variability in axon morphology observed in our model. In particular, mechanistic constraints imposed by other *γ* neurons growing synchronously and competing for space define final axon paths and impact on the formation of branches. Remarkably, previous work has shown that, like mammalian brain structures, MBs exhibit a unique degree of flexibility in their organization, with neurons establishing plastic synapses and receiving unstructured rather than stereotyped inputs [48–50]. Thus, establishing a dense network of non-stereotypic axonal branches may be an optimal strategy for MB *γ* neurons to perform their described integration function, and in particular contextualize novel sensory experiences to provide adapted output behavior [51, 52].

### Axon branching facilitates axon elongation

An interesting finding of our work is that *γ* axons forming side branches grow more efficiently than axons unable to branch. Indeed, populations of branching axons reach the end of the medial lobe with a higher overall frequency than non-branching axons. Furthermore, populations of branching axons also complete overall growth more rapidly (half the time to complete growth at 95% compared to non-branching axons), which could be beneficial in the context of maturing brains subjected to the timing constraints of developmental programs. The importance of branching is also visible at the single cell level, as simulating the growth of individual non-branching axons in a competition context revealed that axons unable to form branches are out-competed by branching neighbors. This prediction fits with the growth defects observed in single *imp* mutant neurons grown *in* *vivo* in a wild-type environment.

How does axon branching promote elongation? The main advantage of forming branches is likely to provide neurons with the possibility to overcome local mechanical hindrances preventing the growth of axonal processes by re-deploying their axonal trees into less spatially constraint regions. Such a process may be compared to the “selective branching” model proposed to explain the oriented growth of terminal axonal arbors in response to guidance cues [53, 54]. In both cases, forming branches that can optimally respond to external cues, and thus exhibit preferential growth, represents an efficient means to regulate the elongation of axon arbors, and to adapt it to variations of the local chemical or mechanical environment.

### Importance of axon-axon interactions in populations of growing neurons

Another finding of this study is the importance of axon-axon mechanical interactions when considering *in* *vivo* axonal growth. Such interactions were shown in different contexts to underlie the establishment of functional neuronal architecture, by promoting the self-organization of axonal arrays and their incorporation into interconnected fibers and circuits, or by defining adapted target innervation patterns [15]. Axon-axon fasciculation, for example, promotes the sorting of axons into bundles, thus facilitating the coordinated long-range navigation of axon populations [15, 55]. Axon-axon repulsion, in contrast, is essential in tiling strategies, where branches must maximize the surface they cover and minimize overlap between neighboring arbors [32, 56]. In *Drosophila* MBs, *γ* axons de-fasciculate when leaving the peduncle, and then innervate the medial lobe at high density, filling it with projections [34, 57]. Axons growing in such a crowded environment thus compete with others for space, and must evolve strategies to grow optimally.

Here, we have shown that axon branching in response to mechanical obstacles is more favorable to overall axon elongation than random branching. Indeed, forming new branches “on demand” may be an optimal strategy to provide responses adapted to local spatial constrains while preventing crowding of the growth space. Of note, we have here considered that the limits of the growth cavity and the neighboring growing axons represent the main source of mechanical obstacles, but the presence of connecting neurons extending their processes to reach *γ* axons is likely to also play a role. Adult *γ* axons, indeed, establish synapses with distinct populations of MB output neurons along the medial lobe, and also receive direct inputs from specific groups of modulatory neurons [34, 58, 59]. Thus, a possibility is that the increased density of branch points observed in the central part of the lobe (Fig 5B) may also reflect the presence of a high density of afferent and efferent neurons extending branches in this region. In the future, it will be interesting to implement in the model the presence of external processes extending to connect to *γ* neurons.

To date, the molecular and cellular mechanisms that may trigger formation of new branches in response to mechanical hindrance are unknown, but a possibility is that branching may be induced by axon growth cone pausing. Indeed, real-time imaging of cultured sensorimotor neurons has revealed that new branches were formed at sites where growth cones had paused, shortly after they resumed their growth [60]. Pausing was proposed to enable accumulation of material such as cytoskeletal components required for branch initiation [61]. An alternative hypothesis is that branching may be triggered by a branch-promoting signaling induced upon contact with neighboring neurons. Such a signal may be mediated by transmembrane signaling molecules present at the surface of neighboring cells or *via* synaptogenesis, as both were shown to induce branch formation [62–64].

In conclusion, the new principles that emerged from our model may underlie the growth and functionality of various populations of axons. For example, cerebellum-like structures, sometimes described as the vertebrate counterparts of MBs, also consist of large collections of equivalent cells that project axon-like processes into densely-packed parallel fibers [65, 66]. In the future, it will also be interesting to apply our model to vertebrate brain interconnected structures with different neuro-architectures, and to explore its relevance during both developmental and regenerative axon growth.

## Methods

### Generation and confocal imaging of MARCM clones

MARCM clones were generated as described by Wu and Luo [67], using the following fly stocks: hsp-flp, tub-Gal80, FRT19A; 201YGal4,UAScGFP; FRT19A and FRT19A *imp*^7^. Brains were dissected at the adult stage, and stained with anti-GFP (molecular probes life technology; ref A11122) and anti-FasciclinII (1D4, DSHB) primary antibodies, revealed by respectively anti-rabbit Alexa 546 and anti-mouse Cy5 secondary antibodies (see [41] for a detailed procedure). Brains were mounted in propyl-galate mounting medium, and imaged with an inverted Zeiss LSM 710 confocal microscope equipped with a 40X/1.1 NA water objective. Z sections were taken every 0.6 to 0.9 *μ*m, with a xy pixel size of 0.09 *μ*m.

### Simulations

Axons continue growing if i) they have not reached the end of the medial lobe, ii) their counter is smaller or equal to a fixed maximum value, and, iii) no other type I branch from the same neuron has reached the extremity of the medial lobe. The value of the counter is incremented by two at each time point *t*_*j*_ if the axon fails to elongate *n*_*max*_ steps. Axons finding too many mechanical obstacles along their way will thus reach the maximum counter value before reaching the lobe extremity. The simulation is completed when there is no growing axon left.

Branching may occur at every time point *t*_*j*_ after elongation. Type I branch origin can be random (with uniform probability *P*_*b*_) or upon contact (after encountering two mechanical constraints in the same time point). In the first case (random branching), the branching point is placed at a step performed during *t*_*j*_ and selected randomly, while in the second one (branching upon contact) it is placed at the axon tip. We estimated the Poisson parameter λ_*b*_ based on the distances between branching points in our data set. A type I branch (random or upon contact) effectively emerges if a random uniform number from zero to one is less or equal than the value of the Poisson probability for the distance from the tip to the nearest branching point. To place a type II branch, a random distance is drawn from the Poisson distribution. If it fits (*rand*_*Poisson*_ ≤ *DPB* (Distance to the Previous Branch)) the branch is placed at that distance from the last branch. Both types of branches initially emerge with a random uniform angle. Type II branches measure between 2 and 10 *μ*m, and appear and disappear randomly during all the simulation until they contact another branch tip or branching point and get stabilized. If they do not stabilize by the end of the simulation they get lost. Type I branches grow following the same rules as main axons.

Main axons were automatically defined using a previously described algorithm ([42] and Supporting information). For visualization, main axon length were projected to the xy plane (in 2D) to avoid the bias due to the low resolution of confocal images in z, and to the compression of the sample along the z axis. 3D length distributions are shown in S7A and S8A Figs. The simulation code is written in MATLAB and is provided as an annotated source code.

## Supporting information

S1 Text Supporting methods. (PDF)

S1 Fig Influence of *α* and *β* values on axon trajectories. *α* represents the axon rigidity; values near zero (<< 1) result in very tortuous trajectories, while higher values (>> 10) lead to the formation of straight axons. *β* represents the axon sensitivity to the external field (represented by the yellow arrow). Values near zero (<< 1) indicate no perception of the field direction, leading to lost axons. Axons with high *β* (>> 10) follow with high fidelity the field direction (in this case, straight). Scale Bar: 10 *μ*m.
(EPS)

S2 Fig Influence of other key parameters on axon properties. (A) Influence of the total number of axons (*Num*_*ax*_) on axon elongation. (B) Average number of branches per axon in function of the branching probability *P*_*b*_ for random branches, and different axonal diameter values (*d*). (C) Influence of total growth duration on axon elongation (counter_max_). For all the experiments, the error bars represent the standard deviation (n = 3 simulations). The following default parameter values were used: ***α*** = 9 and ***β*** = 2, **Δ*ρ*** = 1, ***ψ*** = 0 rad, ***ω***: uniform in all the space, **λ**_***b***_ = 15, ***b***_***l***_ = 1, ***d*** = 0.4 *μ*m, ***Num***_***ax***_ = 400, ***n***_***max***_ = 6, ***n***_***r***_ = 2, **counter**_**max**_ = 140, ***X***_***max***_ = 70, ***ξ***: cylinder of radius 13 *μ*m and ***P***_***b***_ = 0.2.
(EPS)

S3 Fig Generation of axon reconstructions from confocal 3D images: Description of the database. (A) Maximum intensity projection of a confocal image depicting a single *γ* neuron stained with GFP (in white, top), its skeleton after segmentation (in red, middle) and the overlay between the original axon and its reconstructed skeleton (bottom). (B) Standard medial lobe used for the registration of individual axons to a reference lobe. The Fasciclin II staining is used to visualize the entry into the lobe (red line), as well as the lobe extremity (green line). The yellow dot depicts the coordinate origin and the blue dotted line shows the axis used to rotate all the axons. Scale bar: 10 *μ*m. (C) Database of the 43 reconstructed wild-type *γ* axons. (D) Collection of wild-type *γ* axons reconstructed from original confocal images and placed together in a reference medial lobe. Individual axons were labeled with distinct false-colors for visualization (n = 43). (E) Local mean directionality (red arrows) of *γ* axons along the medial lobe (2D projection). The lobe was divided into rectangular parallelepipeds, and the mean directionality of all the axonal segments included in this volume was calculated. The dotted line on the right represents the midline.
(TIF)

S4 Fig Characterization of type II branch length and dynamics using live-imaging of growing *γ* axons. (A) Image sequence extracted from S1 Video, where a single neuron from a wild-type brain undergoing metamorphosis is shown over three time points (15 min total), during the regrowth phase. Short (type II) and long (type I) branches are highlighted with light blue asterisk and purple arrows, respectively. Light blue asterisks highlight the same type II branches over three consecutive time points, revealing the dynamicity of these branches. Scale Bar: 5 *μ*m. (B) Frequency distribution of Type II branch length measured as described in the Supporting information.
(EPS)

S5 Fig Parameter estimation from *in vivo* data. (A) Frequency distributions of parameters estimated from real axons (top), or obtained for individual Markov Chains (middle) and individual Markov Chains with discontinuities (bottom). The p values are calculated using a Kruskal Wallis test. (B-B”) Parameter estimation functions. (B) Addition of the p values comparing the distributions from real data with simulated axons with discontinuities, in function of *α* and *β*. (B’) Polynomial surface that approximates the function in (B). The black square indicates the pair of parameters that maximizes the surface. (B”) Parameter estimation surfaces for two random halves of real samples. The black square indicates the pair of parameters that maximizes the surface for the entire population (the same as in (B’)), and the smaller grey ones the values that maximize the surfaces corresponding to each random half of the population. The simulations were done considering an entire *γ* population of 650 neurons and type I branching upon contact.
(EPS)

S6 Fig Estimation of attractive field, medial lobe geometry and axon diameter. (A) Schematic representation of the *D* and *V* distances used to generate different attractive field configurations. The attractive source is positioned at the end of the medial lobe, and depends on two variables (*D*, *V*). *D* describes the extent of the source along the dorsal part of the lobe, and *V* its extent along the ventral part. The origin point (*D*, *V*) = (0, 0) is also shown. (B) Attractive field configuration used for the simulations. The blue color code is used to illustrate the orientation of the gradient along the lobe, and the yellow color depicts the inverted C shape of the source. (C) Similarity index in function of *D* and *V*. The attractive field configuration used in this study ((*D*, *V*) = (45, 30)) is represented by a black dot. Its similarity value is 0.779. This index is represented as a heat map (yellow: highest similarity and blue: lowest similarity). (D) Percentage of axons failing to complete their growth in simulations considering different plausible diameter sizes. The simulations were done considering an entire *γ* population of 650 neurons and type I branching upon contact. The diameter value used in the model is the highest for a percentage of non-elongated axons lower than 5%. (E) 3D geometry of the medial lobe used in the simulations. Axons start their growth perpendicular to the purple plane, representing the peduncle transversal section, and their growth is constrained to the volume delimited by surfaces represented by the blue lines.
(EPS)

S7 Fig Simulated *γ* axon morphologies: Random versus mechanical branching. (A) Frequency distributions of 3D main axon lengths from real and simulated axons. Data from real wild-type *γ* axons are shown in the upper panels (n = 43), and data from simulated ones in the lower panels (n = 650). (B,C): Frequency distributions of inter-axon distances reflecting the similarity between simulated and real axons (B) and the intra-group variability (C). Inter-axon distances were calculated with the ESA distance [40]. In grey, Real *vs* real; in light green, Real vs Simu and dark green represents the overlap of the two histograms. Values shown in these graphs correspond to those displayed in Fig 5D and 5E.
(EPS)

S8 Fig Simulations of single non-branching axons: Comparison with real data. (A) Frequency distributions of 3D main axon lengths from real and simulated axons. Data from simulated single non-branching axons are shown in the upper panels (n = 45), and data from real single *imp* mutant *γ* axons in the lower panels (n = 45). (B) Frequency distributions of inter-axon distances reflecting the similarity between simulated and real *imp* mutant axons. Inter-axon distances were calculated with the ESA distance [40]. In grey, Real vs real; in light green, Real vs Simu and dark green represents the overlap of the two histograms. (C) Measure of similarity between real *imp* mutant and simulated axons. Boxplots of inter-axon distances (measured with the ESA distance [40] within the population of real *imp* mutant axons (real, n = 45), and between real *imp* mutant and simulated axons.
(EPS)

S9 Fig Influence of apparent growth velocity (*n*_*max*_) on axon elongation. (A) Percentage of individual non-branching axons failing to elongate in function of *n*_*max*_. 40 non-branching axons growing together with 360 branching axons were simulated (*Num*_*ax*_ = 400). While the speed (*n*_*max*_) of branching axons was left constant (*n*_*max*_ = 6), increasing values were used for non-branching axons. The error bars represent the standard deviation (n = 3 simulations). (B) Influence of *n*_*max*_ on a homogeneous population of growing axons. In these simulations, all axons were branching. The following parameter values were used: ***α*** = 9 and ***β*** = 2, **Δ*ρ*** = 1, ***ψ*** = 0 rad, ***ω***: uniform in all the space, **λ**_***b***_ = 15, ***b***_***l***_ = 1, ***d*** = 0.4 *μ*m, ***n***_***r***_ = 2, **counter**_**max**_ = 140, ***X***_***max***_ = 70, ***ξ***: cylinder of radius 13 *μ*m and ***P***_***b***_ = 0.2.
(EPS)

S1 Video Single wild-type *γ* axon growing in the medial lobe of a pupal brain (30h APF stage). The recorded *γ* neurons was labeled with GFP using the MARCM technique (See Methods and Medioni et al., 2015 Nat Protoc. 2015 Apr;10(4):574-84. doi: 10.1038/nprot.2015.034. Epub 2015 Mar 12). Scale Bar: 5 *μ*m. Time lag: 5 min. Genotype: hsflp122, tub GAL-80; FRT19A; 201Y GAL4, UAS cGFP.
(AVI)

S1 Folder Original scripts and raw data. (ZIP)
