# Supplementary material for: A stochastic framework to model axon interactions within growing neuronal populations
Source: PLoS Comput Biol. 2018 Dec 3;14(12):e1006627. doi: 10.1371/journal.pcbi.1006627 (PMC6292646; doi:10.1371/journal.pcbi.1006627)
Supplement: S1 Text — (PDF) [file pcbi.1006627.s001.pdf]

Supporting information

S1 text: Supporting Methods

Generation of confocal images of entire populations of γ neurons. Confocal images shown in Fig. 4 A were obtained using the 10x 0.6 NA air objective of a LSM780 NLO Zeiss microscope. Z sections were taken every 0.5 μm, with a xy pixel size of 0.57 μm. 3D projections have been created using FiJi [1]. The images used for the reconstruction shown in Fig. 4 B were obtained using the 40X 1.2 NA water objective and the pulsed laser of a LSM780 NLO Zeiss microscope. Z sections were taken every 0.4 μm, with a xy pixel size of 0.11 μm. The 3D reconstruction was generated using the surface mode of Imaris 8.1 Bitplane software.

Reconstruction of individual axons and representation in a reference lobe. The pixel size of the confocal images was made isotropic by the bicubic interpolation in FiJi [1]. To extract the morphologies of individual axons from the 3D confocal images, we reconstructed axonal trees using the Neuromantic software [2], and obtained the euclidean coordinates of all the points describing the axon. The curves were re-sampled by segments of 1 μm ($\Delta\rho = 1$ μm) to be coherent with the general axonal diameter in the images. So-called main axons were automatically defined using an algorithm that takes into account the length, directionality and sense coherence of each curve in the tree [3].

Confocal images of our dataset were acquired from different brains with lobes of slightly different geometry and dimensions. To align all reconstructed axons into the same virtual medial lobe, we chose a reference lobe in our database, and normalized axon length to medial lobe length. Two main curves, signaling the beginning of the medial lobe and its end were drawn based on FasciclinII staining (red and green lines respectively in S3 B Fig). We then extracted the lobe entrance and end points of each axon and corrected total axon length according to the quotient of the distance between the entry and the end point in the particular image and in the reference lobe. Every neuron was also rotated to place the blue dotted line in S3 B Fig parallel to the horizontal axis. We chose this procedure instead of a classic image registration method because a rigid transformation would not take into account differences in the morphology of the lobes, while a non-rigid transformation would deform the axon morphology.

Model renormalization. We can calculate $P(\theta_i|\theta_{i-2})$ knowing $P(\theta_i|\theta_{i-1})$ (*i.e.* renormalization by decimation) as

$$P(\theta_i|\theta_{i-2}) = \int_{-\infty}^{\infty} P(\theta_i|\theta_{i-1})P(\theta_{i-1}|\theta_{i-2})d_{\theta_{i-1}} \tag{1}$$

to obtain the expression

$$P(\theta_i|\theta_{i-2}) \propto exp - [\alpha'(\theta_i - \theta_{i-2})^2 + \beta'(\theta_i)^2], \tag{2}$$

with

$$\alpha' = f_1(\alpha, \beta) = \frac{(\alpha+\beta)\alpha^2}{(\alpha+\beta)^2+\alpha^2} \tag{3}$$

$$\beta' = f_2(\alpha, \beta) = \frac{(\alpha+\beta)[(\alpha+\beta)^2 - \alpha^2]}{(\alpha+\beta)^2 + \alpha^2}. \tag{4}$$

Thus, α' and β' are the parameters defining the probability of θ_i conditioned by θ_{i-2}[1]. Interestingly, these equations are invertible such as

$$\alpha = f_1^{-1}(\alpha', \beta') = \alpha'' = \sqrt{\frac{(2\alpha'+\beta')^3 - (2\alpha'+\beta')^2(\alpha'+\beta')}{\alpha'+\beta'}} \tag{5}$$

$$\beta = f_2^{-1}(\alpha', \beta') = \beta'' = 2\alpha' + \beta' - \alpha''. \tag{6}$$

Thus, we can extrapolate the values of the parameters from the current scale to any other, including a smaller one (*e.g.* to have two or more data points instead of one), to compute the equivalent models to a certain scale to any other.

Proof of Eq. 5. By the model definition, we have

$$\theta_i = \frac{\alpha}{\alpha+\beta}\theta_{i-1} + \xi_i, \tag{7}$$

where

$$\xi_i \sim \mathcal{N}(0, \frac{1}{2(\alpha+\beta)}). \tag{8}$$

If we denote $\gamma = \frac{\alpha}{\alpha+\beta}$, we then have

$$\theta_{i+1} = \gamma\theta_i + \xi_{i+1} \tag{9}$$

$$\theta_{i+1} = \gamma[\gamma\theta_{i-1} + \xi_i] + \xi_{i+1} \tag{10}$$

$$\theta_{i+1} = \gamma^2\theta_{i-1} + \gamma\xi_i + \xi_{i+1}, \tag{11}$$

where ξ_i and ξ_{i+1} are two independent random variables with the same Gaussian distribution (Eq. 8). Therefore, denoting $\sigma_0^2 = \frac{1}{2(\alpha+\beta)}$;

$$\gamma\xi_i =\sim \mathcal{N}(0, \gamma^2\sigma_0^2), \tag{12}$$

and

$$\gamma\xi_i + \xi_{i+1} = \mathcal{N}(0, (\gamma^2+1)\sigma_0^2). \tag{13}$$

Finally,

$$\theta_{i+1} = \gamma^2\theta_{i-1} + \tau_{i+1}, \tag{14}$$

where $\tau_{i+1} \sim \mathcal{N}(0, (\gamma^2+1)\sigma_0^2)$.
Similarly,

$$\theta_{i+2} = \gamma\theta_{i+1} + \xi_{i+2}, \tag{15}$$

where $\xi_{i+2} \sim \mathcal{N}(0, \sigma_0^2)$.
Using Eq. 14

$$\theta_{i+2} = \gamma[\gamma^2\theta_{i-1} + \tau_{i+1}] + \xi_{i+2} \tag{16}$$

[1] α' and β' can alternatively be obtained by recursively applying Eq. 3

$$\theta_{i+2} = \gamma^3\theta_{i-1} + \gamma\tau_{i+1} + \xi_{i+2}. \quad (17)$$

τ_{i+1} and ξ_{i+2} are independent, thus

$$\theta_{i+2} = \gamma^3\theta_{i-1} + \rho_{i+2}, \quad (18)$$

with $\rho_{i+2} \sim \mathcal{N}(0, (\gamma^4 + \gamma^2 + 1)\sigma_0^2)$.
By recursion we obtain the cumulated variance as the series

$$\sigma^2_{\theta_{i=1:M}} = \sigma_0^2 \sum_{i=0}^{M-1} \gamma^{2i}. \quad (19)$$

The series in Eq. 19 is geometrical, thus

$$\sigma^2_{\theta_i} = \sigma_0^2 \frac{1-\gamma^{2M}}{1-\gamma^2}. \quad (20)$$

that converges to

$$\sigma^2_{\theta_\infty} = \frac{\sigma_0^2}{1-\gamma^2}. \quad (21)$$

Proof of Eq. 8. The difference $\Delta\theta = \theta_i - \theta_{i-1}$ is equal to

$$\Delta\theta_i = \gamma\theta_{i-1} + \xi_i - \theta_{i-1} = (\gamma - 1)\theta_{i-1} + \xi_i. \quad (22)$$

The variance of $\Delta\theta = \theta_i - \theta_{i-1}$ is thus

$$\sigma^2_{\Delta\theta_i} = (\gamma-1)^2\sigma^2_{\theta_{i-1}} + \sigma_0^2 - 2E(\theta_{i-1}\xi_i). \quad (23)$$

θ_{i-1} and ξ_i are independent and centered in 0, thus the expectation $E(\theta_{i-1}\xi_i) = E(\theta_{i-1})E(\xi_i) = 0$, and with Eq. 21

$$\sigma^2_{\Delta\theta_\infty} = \sigma_0^2[\frac{(\gamma-1)^2}{1-\gamma^2} + 1] = \frac{2\sigma_0^2(1-\gamma)}{1-\gamma^2}. \quad (24)$$

Parameter estimation. We estimated the α and β parameters from each individual axon following eq. 9. The distributions of parameters estimated in the latter case are shown in S5 A Fig (Real data). Surprisingly, these distributions did not follow a Gaussian distribution centered on the average parameters of the whole population. However, when simulating individual Markov chains using the estimated mean parameters and estimating the parameters on these simulated axons, we obtained Gaussian-like distributions (S5 A Fig; Simulated). This difference in distributions between the parameter estimated values of real and simulated axons might be explained by the existence of some broken points in the Markov Chain of real axons. These broken points are generated when axons change trajectory upon encountering an obstacle, preventing a direct estimation from the data. To overcome this problem, we defined a new estimation scheme as follows: 1) we estimated the parameters for each individual axon in the dataset, saved the distributions of estimated parameters and considered the mean value of each parameter; 2) we simulated Markov Chains with a range of parameters values centered on the mean values estimated from real axons, and included random broken points; 3) we estimated the parameters from the resulting simulations and saved the distributions for each tested pair of parameters (see S5 A Fig for an example of such a distribution; Simulated with discontinuities); 4) we selected the pair of parameters whose distribution is most similar to that obtained from the data

using the Kruskal Wallis test. In order to accomplish step 4, we have added the p values of each test for α and β, and obtained the 2D function shown in S5 B Fig. We then approximated this function with a polynomial surface (S5 B' Fig) and chose the pair of parameters for which this surface reaches its maximum value, ($\hat{\alpha} = 7.45$ and $\hat{\beta} = 1.67$).

We designed the attractive vector field as the gradient of a function that is maximal in the region of the highest attraction. To find the (simple) function that better fits our data, we evaluated each candidate with a similarity index, equal to the average of the cosine of the difference between the angle of each segment composing the axons in data with the corresponding field directionality. This index thus goes from -1 (opposite direction) to 1 (perfect match). The function with the maximum score was chosen. The field was calibrated using the angles on the xy plane, and considered constant in z. To systematically test a range of possible configurations, we considered two variables (D, V) accounting for the dorsal and ventral extension of the attractive source (S6 B Fig). The initial point $(D, V) = (0, 0)$ represents a punctual source placed at the center of the lobe extremity. We then varied (D, V) from 0 to 60 μm with intervals of 5 μm and calculated all corresponding similarity indexes.

To implement volume exclusion in our model, at each growth step the axon tip evaluates its distance from every other axon and type I branch in the lobe. A collision is considered if the distance from the tip to any other axonal segment is inferior to the axonal diameter d. We considered the axon volume as a sequence of spheres with their center placed equidistantly through the axon path. The axon diameter d is thus established by the diameter of the spheres, and constant through the axon. Its value was estimated from published electron microscopy images [4], and optimized by simulations considering branching upon contact (see S6 E Fig and Methods). The selected diameter value corresponds to the first elbow of the obtained logistic function.

Live imaging. Brains were dissected out of pupae 24-30 APF (After Pupa Formation) and mounted in a Labtek II chambered coverglass (#155378, Fisher Scientific) in culture medium (Schneider medium, 10% FCS, 1% Antibiotic Antimycotic Solution (Sigma), 200μg/ml insulin (Sigma), 1μg/ml ecdysone (20HE; Sigma) (see [5] for a detailed protocol). Brains were imaged using a Zeiss LSM780 NLO inverted two-photon microscope and a 40X/1.2 NA water objective. Medial lobe regions were imaged. Z sections were taken every 0.8 μm and covered the entire medial lobe volume. Z stacks were acquired every 5 minutes over up to 15 hours. Pixel size is 0.13 μm.

Live imaging drift correction and branch length measurements. Drift correction was inspired by [6]. However, computing 3D affine transformations as in this publication was not possible: first, only single axons were imaged, yielding images with a sparse information which, in turn, may incorrectly be co-registered with an affine transformation; second, the optical slice thickness is larger than the pixel, which amplify this behavior.

We thus performed a two-fold drift compensation. First, maximum intensity (MIP) projections of the acquired images were computed, couple of successive MIPs (that are 2D images) were co-registered with 2D affine transformations, and these transformations were compounded to express all the transformations with respect to a reference image (say the first one). This first step allowed an in-plane drift compensation, but a motion along the z direction may still exist. A residual translation along the z direction was then estimated using the multiple transformation strategy described in [6]. The compounding of the 2D affine transformation and the z translation yielded the final 3D drift compensation.

The segmentation of these 3D+t series proceeded in two steps. First, main axon and type I branches were defined as segments that remain stable throughout the whole

series and were extracted. Their centerlines were computed on the first time points, *via* classical image processing tools (thresholding, skeletonization, and manual selection). Then, they were propagated throughout the whole 3D+t series. The use of homotopy-preserving operations guarantees that the main axon and type I branches form a tree (in a topological sense). Type II branches were then segmented by the means of an homotopic dilatation from main axon and type I branches. Centerlines were extracted and tracked throughout the series, and their maximal length was measured.

Statistical Analysis. To compare populations, we used sample sizes defined such that the power of the tests used for statistical analysis is higher than 90%. Data were not assumed to be normally distributed, and the non-parametric Kruskal-Wallis test was used for all comparisons. All samples were kept for analysis, and outliers were not excluded.

Supporting Figure and Video Legends

S1 Fig. Influence of $\boldsymbol{\alpha}$ and $\boldsymbol{\beta}$ values on axon trajectories. α represents the axon rigidity; values near zero ($<< 1$) result in very tortuous trajectories, while higher values ($>> 10$) lead to the formation of straight axons. β represents the axon sensitivity to the external field (represented by the yellow arrow). Values near zero ($<< 1$) indicate no perception of the field direction, leading to lost axons. Axons with high β ($>> 10$) follow with high fidelity the field direction (in this case, straight). Scale Bar: 10 μm.

S2 Fig. Influence of other key parameters on axon properties. (A) Influence of the total number of axons (Num_{ax}) on axon elongation. (B) Average number of branches per axon in function of the branching probability P_b for random branches, and different axonal diameter values (d). (C) Influence of total growth duration on axon elongation ($\mathrm{counter_{max}}$). For all the experiments, the error bars represent the standard deviation (n = 3 simulations). The following default parameter values were used: $\boldsymbol{\alpha} = 9$ and $\boldsymbol{\beta} = 2$, $\boldsymbol{\Delta\rho} = 1$, $\boldsymbol{\psi} = 0$ rad, $\boldsymbol{\omega}$: uniform in all the space, $\boldsymbol{\lambda_b} = 15$, $\boldsymbol{b_l} = 1$, $\boldsymbol{d} = 0.4$ μm, $\boldsymbol{Num_{ax}} = 400$, $\boldsymbol{n_{max}} = 6$, $\boldsymbol{n_r} = 2$, $\mathbf{counter_{max}}$=140, $\boldsymbol{X_{max}} = 70$, $\boldsymbol{\xi}$: cylinder of radius 13 μm and $\boldsymbol{P_b} = 0.2$.

S3 Fig. Generation of axon reconstructions from confocal 3D images: description of the database. (A) Maximum intensity projection of a confocal image depicting a single γ neuron stained with GFP (in white, top), its skeleton after segmentation (in red, middle) and the overlay between the original axon and its reconstructed skeleton (bottom). (B) Standard medial lobe used for the registration of individual axons to a reference lobe. The Fasciclin II staining is used to visualize the entry into the lobe (red line), as well as the lobe extremity (green line). The yellow dot depicts the coordinate origin and the blue dotted line shows the axis used to rotate all the axons. Scale bar: 10 μm. (C) Database of the 43 reconstructed wild-type γ axons. (D) Collection of wild-type γ axons reconstructed from original confocal images and placed together in a reference medial lobe. Individual axons were labeled with distinct false-colors for visualization (n = 43). (E) Local mean directionality (red arrows) of γ axons along the medial lobe (2D projection). The lobe was divided into rectangular parallelepipeds, and the mean directionality of all the axonal segments included in this volume was calculated. The dotted line on the right represents the midline.

S4 Fig. Characterization of type II branch length and dynamics using live-imaging of growing γ axons. (A) Image sequence extracted from S1 Video, where a single neuron from a wild-type brain undergoing metamorphosis is shown over three time points (15 min total), during the regrowth phase. Short (type II) and long (type I) branches are highlighted with light blue asterisk and purple arrows, respectively. Light blue asterisks highlight the same type II branches over three consecutive time points, revealing the dynamicity of these branches. Scale Bar: 5 μm. (B) Frequency distribution of Type II branch length measured as described in the Supporting information.

S5 Fig. Parameter estimation from *in vivo* data. (A) Frequency distributions of parameters estimated from real axons (D), or obtained for individual Markov Chains and individual Markov Chains with discontinuities. The p values are calculated using a Kruskal Wallis test. (B-B") Parameter estimation functions. (B) Addition of the p values comparing the distributions from real data with simulated axons with discontinuities, in function of α and β. B': Polynomial surface that approximates the function in (B). The black square indicates the pair of parameters that maximizes the surface. B": Parameter estimation surfaces for two random halves of real samples. The black square indicates the pair of parameters that maximizes the surface for the entire population (the same as in (B')), and the smaller grey ones the values that maximize the surfaces corresponding to each random half of the population. The simulations were done considering an entire γ population of 650 neurons and type I branching upon contact.

S6 Fig. Estimation of attractive field, medial lobe geometry and axon diameter. (A) Schematic representation of the D and V distances used to generate different attractive field configurations. The attractive source is positioned at the end of the medial lobe, and depends on two variables (D, V). D describes the extent of the source along the dorsal part of the lobe, and V its extent along the ventral part. The origin point $(D, V) = (0, 0)$ is also shown. (B) Attractive field configuration used for the simulations. The blue color code is used to illustrate the orientation of the gradient along the lobe, and the yellow color depicts the inverted C shape of the source. (C) Similarity index in function of D and V . The attractive field configuration used in this study ($(D, V) = (45, 30)$) is represented by a black dot. Its similarity value is 0.779. This index is represented as a heat map (yellow: highest similarity and blue: lowest similarity). (D) Percentage of axons failing to complete their growth in simulations considering different plausible diameter sizes. The simulations were done considering an entire γ population of 650 neurons and type I branching upon contact. The diameter value used in the model is the highest for a percentage of non-elongated axons lower than 5%. (E) 3D geometry of the medial lobe used in the simulations. Axons start their growth perpendicular to the purple plane, representing the peduncle transversal section, and their growth is constrained to the volume delimited by surfaces represented by the blue lines.

S7 Fig. Simulated γ axon morphologies: random versus mechanical branching. (A) Frequency distributions of 3D main axon lengths from real and simulated axons. Data from real wild-type γ axons are shown in the upper panels (n = 43), and data from simulated ones in the lower panels (n = 650). (B,C): Frequency distributions of inter-axon distances reflecting the similarity between simulated and real axons (B) and the intra-group variability (C). Inter-axon distances were calculated with the ESA distance [7]. In grey, Real *vs* real; in light green, Real vs Simu and dark green represents the overlap of the two histograms. Values shown in these graphs correspond to those displayed in Figs 5 D and 5 E.

S8 Fig. Simulations of single non-branching axons: comparison with real data. (A) Frequency distributions of 3D main axon lengths from real and simulated axons. Data from simulated single non-branching axons are shown in the upper panels (n = 45), and data from real single *imp* mutant γ axons in the lower panels (n = 45). (B) Frequency distributions of inter-axon distances reflecting the similarity between simulated and real *imp* mutant axons. Inter-axon distances were calculated with the ESA distance [7]. In grey, Real vs real; in light green, Real vs Simu and dark green represents the overlap of the two histograms. (C) Measure of similarity between real *imp* mutant and simulated axons. Boxplots of inter-axon distances (measured with the ESA distance [7] within the population of real *imp* mutant axons (real, n = 45), and between real *imp* mutant and simulated axons.

S9 Fig. Influence of apparent growth velocity (n_{max}) on axon elongation. (A) Percentage of individual non-branching axons failing to elongate in function of n_{max}. 40 non-branching axons growing together with 360 branching axons were simulated (Num_{ax} = 400). While the speed (n_{max}) of branching axons was left constant (n_{max} = 6), increasing values were used for non-branching axons. The error bars represent the standard deviation (n = 3 simulations). (B) Influence of n_{max} on a homogeneous population of growing axons. In these simulations, all axons were branching. The following parameter values were used: $\boldsymbol{\alpha} = 9$ and $\boldsymbol{\beta} = 2$, $\boldsymbol{\Delta\rho} = 1$, $\boldsymbol{\psi} = 0$ rad, $\boldsymbol{\omega}$: uniform in all the space, $\boldsymbol{\lambda_b} = 15$, $\boldsymbol{b_l} = 1$, $\boldsymbol{d} = 0.4$ μm, $\boldsymbol{n_r} = 2$, $\mathbf{counter_{max}} = 140$, $\boldsymbol{X_{max}} = 70$, $\boldsymbol{\xi}$: cylinder of radius 13 μm and $\boldsymbol{P_b} = 0.2$.

S1 Video. Single wild-type γ axon growing in the medial lobe of a pupal brain (30h APF stage). The recorded γ neurons was labeled with GFP using the MARCM technique (See Methods). Scale Bar: 5 μm . Time lag: 5 min. Genotype: hsflp122, tub GAL-80; FRT19A; 201Y GAL4, UAS cGFP.

References

1. Schindelin J, Arganda-Carreras I, Frise E, Kaynig V, Longair M, Pietzsch T, et al. Fiji: an open-source platform for biological-image analysis. Nature methods. 2012;9(7):676–682.

2. Myatt DR, Hadlington T, Ascoli GA, Nasuto SJ. Neuromantic - from semi-manual to semi-automatic reconstruction of neuron morphology. Frontiers in Neuroinformatics. 2012;6:6–4.

3. Razetti A, Descombes X, Medioni C, Besse F. A Stochastic Framework for Neuronal Morphological Comparison: Application to the Study of imp Knockdown Effects in Drosophila Gamma Neurons. In: International Joint Conference on Biomedical Engineering Systems and Technologies. Springer; 2016. p. 145–166.

4. Watts RJ, Schuldiner O, Perrino J, Larsen C, Luo L. Glia engulf degenerating axons during developmental axon pruning. Current Biology. 2004;14(8):678–84.

5. Medioni C, Ephrussi A, Besse F. Live imaging of axonal transport in Drosophila pupal brain explants. Nature Protocols. 2015;10(4):574–84.

6. Medioni C, Besse F, Descombes X, Malandain G. Motion compensation in two-photon microscopy temporal series. In: International Symposium on Biomedical Imaging (ISBI). Brooklyn, United States; 2015. p. 4. Available from: https://hal.inria.fr/hal-01311755.

7. Mottini A, Descombes X, Besse F. From curves to trees: A tree-like shapes distance using the elastic shape analysis framework. Neuroinformatics. 2015;13(2):175–191.
