# Supplementary figures and images for: A stochastic framework to model axon interactions within growing neuronal populations
Source: PLoS Comput Biol. 2018 Dec 3;14(12):e1006627. doi: 10.1371/journal.pcbi.1006627 (PMC6292646; doi:10.1371/journal.pcbi.1006627)

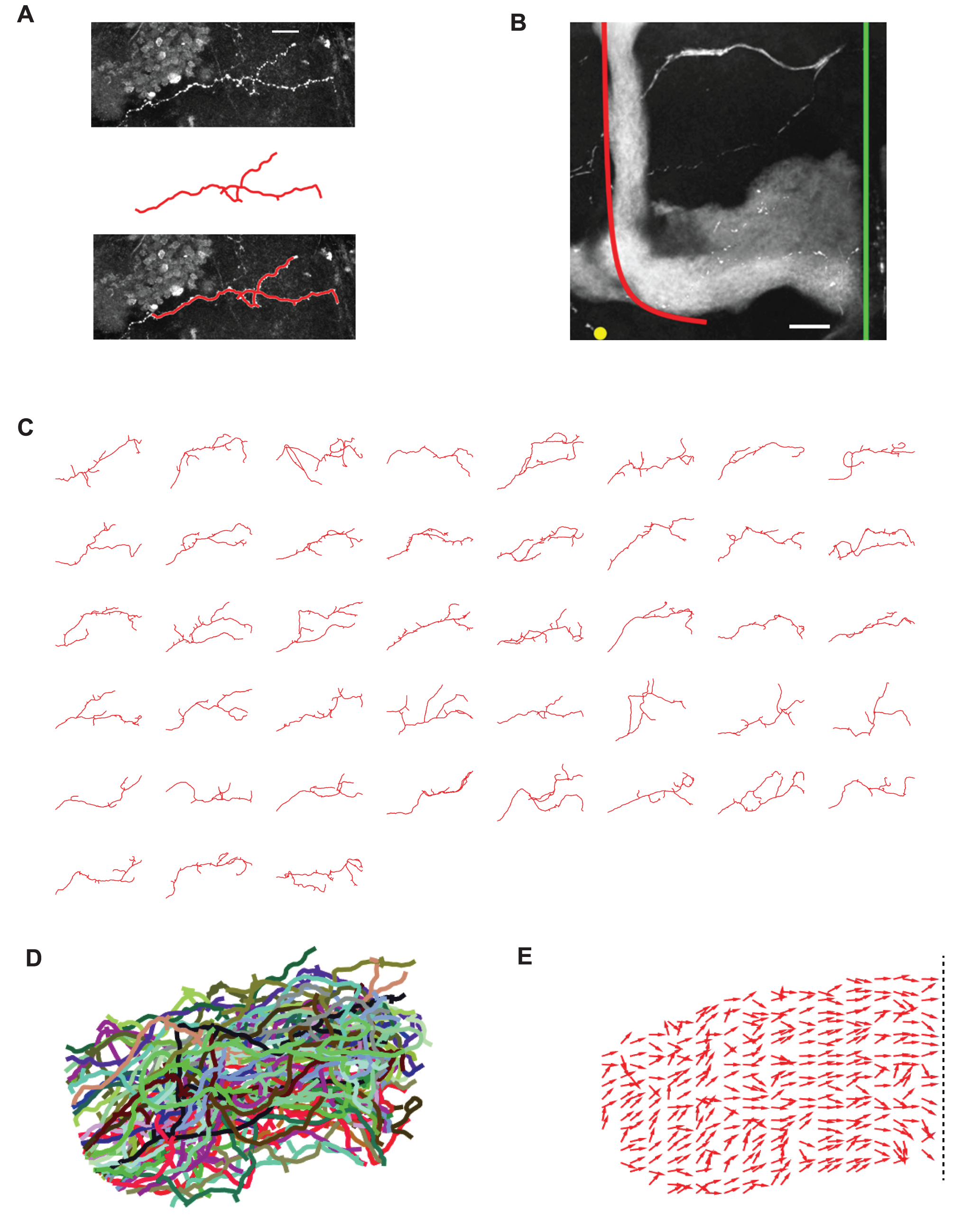

Supplement: S3 Fig — (A) Maximum intensity projection of a confocal image depicting a single γ neuron stained with GFP (in white, top), its skeleton after segmentation (in red, middle) and the overlay between the original axon and its reconstructed skeleton (bottom). (B) Standard medial lobe used for the registration of individual axons to a reference lobe. The Fasciclin II staining is used to visualize the entry into the lobe (red line), as well as the lobe extremity (green line). The yellow dot depicts the coordinate origin and the blue dotted line shows the axis used to rotate all the axons. Scale bar: 10 μm. (C) Database of the 43 reconstructed wild-type γ axons. (D) Collection of wild-type γ axons reconstructed from original confocal images and placed together in a reference medial lobe. Individual axons were labeled with distinct false-colors for visualization (n = 43). (E) Local mean directionality (red arrows) of γ axons along the medial lobe (2D projection). The lobe was divided into rectangular parallelepipeds, and the mean directionality of all the axonal segments included in this volume was calculated. The dotted line on the right represents the midline. (TIF) [file pcbi.1006627.s004.tif]

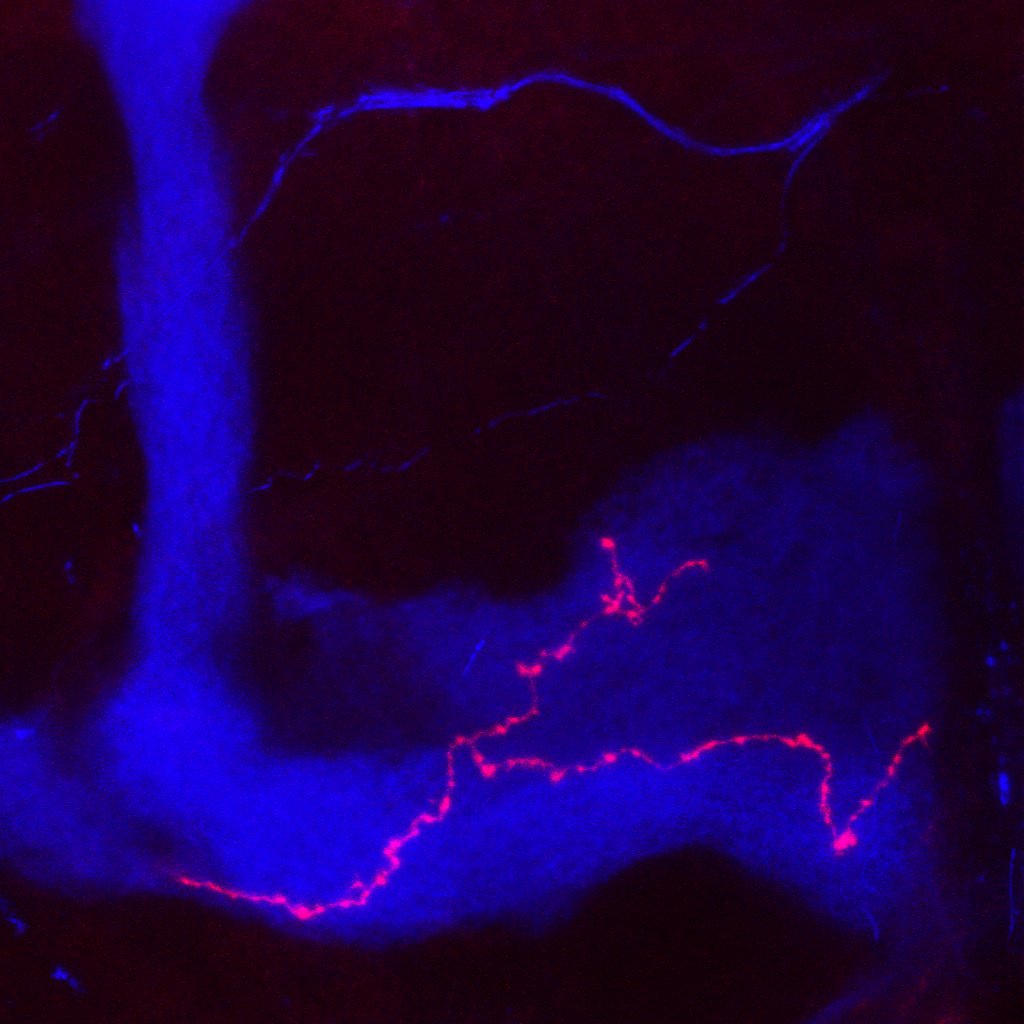

Supplement: S1 Folder — (ZIP) [file pcbi.1006627.s012.zip › Razetti_et_al_scripts/LOBE2.jpg]

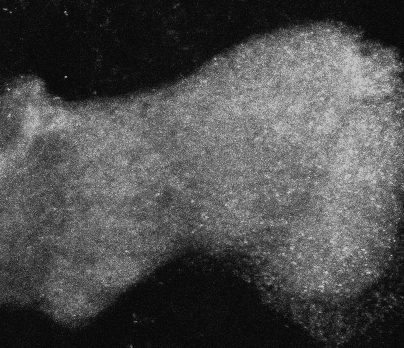

Supplement: S1 Folder — (ZIP) [file pcbi.1006627.s012.zip › Razetti_et_al_scripts/NEW_LOBE.tif]
